# Supplementary material for: Supramolecular metal-organic frameworks that display high homogeneous and heterogeneous photocatalytic activity for H2 production
Source: Nat Commun. 2016 May 10;7:11580. doi: 10.1038/ncomms11580 (PMC4866394; doi:10.1038/ncomms11580)
Supplement: Supplementary Information — Supplementary Figures 1-43, Supplementary Tables 1-3, Supplementary Methods and Supplementary References. [file ncomms11580-s1.pdf]

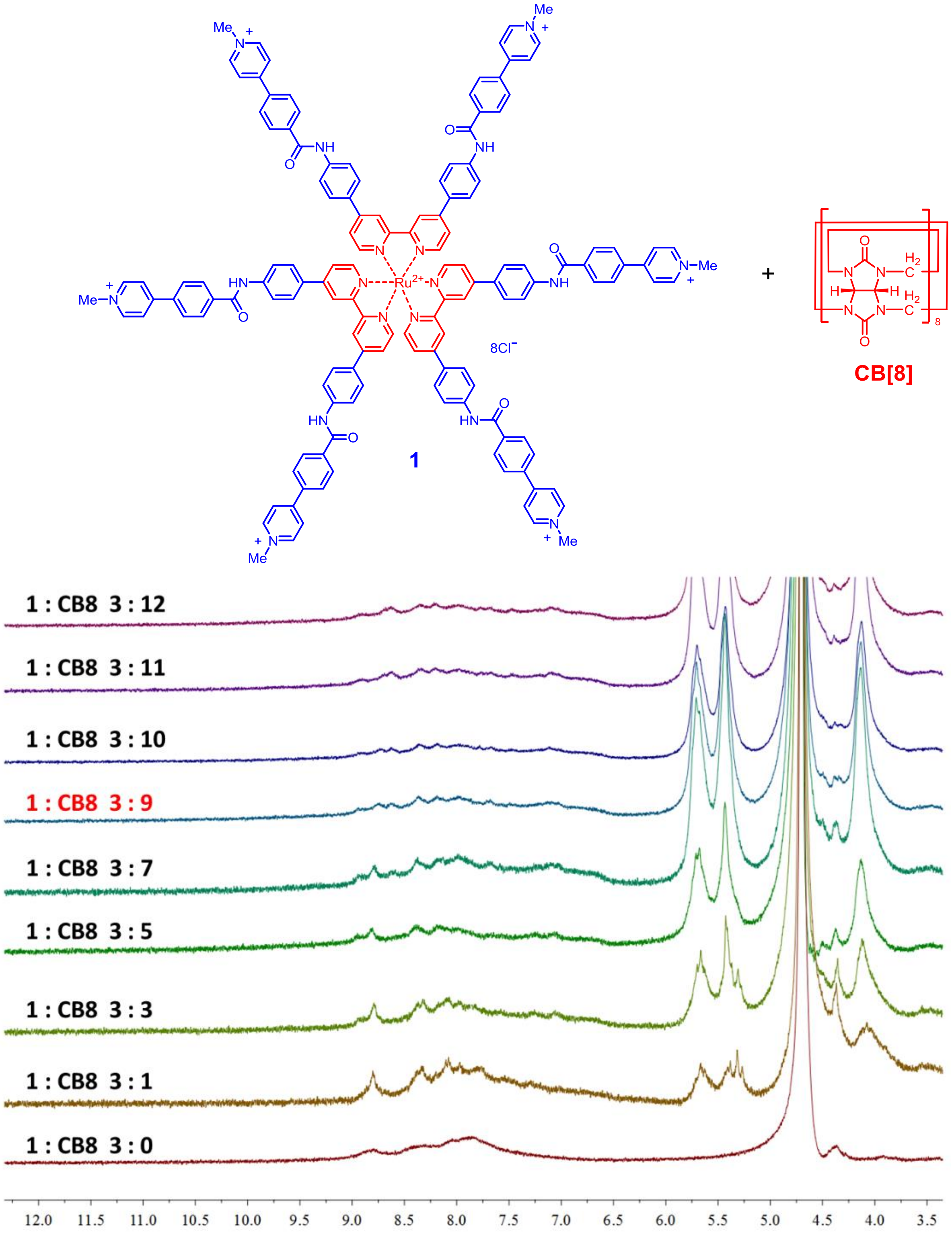

Supplementary Figure 1. ^{1}H NMR titration of **1** in D_2O with increasing amounts of CB[8] (400 MHz, 25 °C, [**1**] = 1.0 mM).

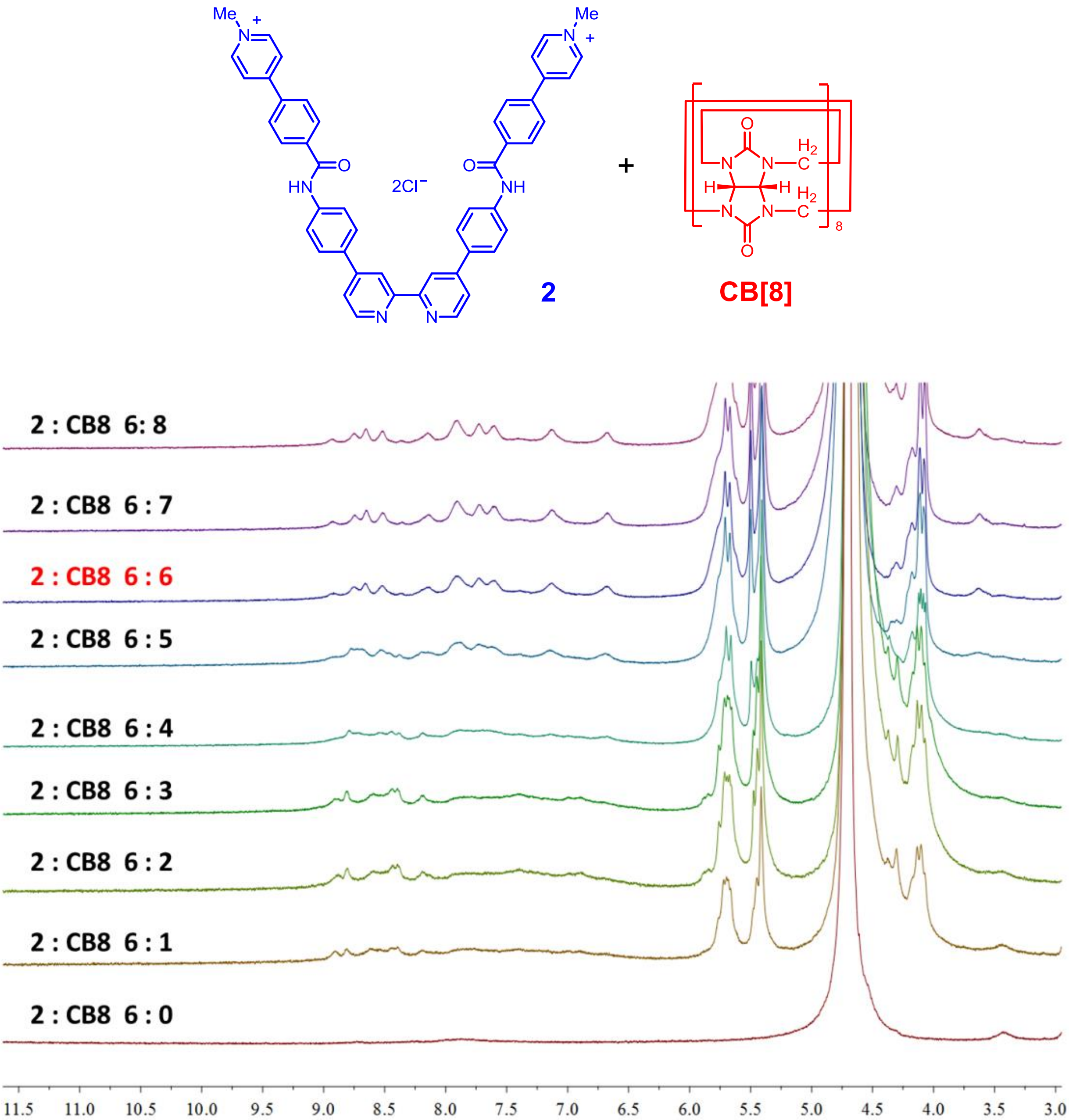

Supplementary Figure 2. ^{1}H NMR titration of **2** in D_2O with increasing amounts of CB[8] (400 MHz, 25 °C, [**2**] = 1.0 mM).

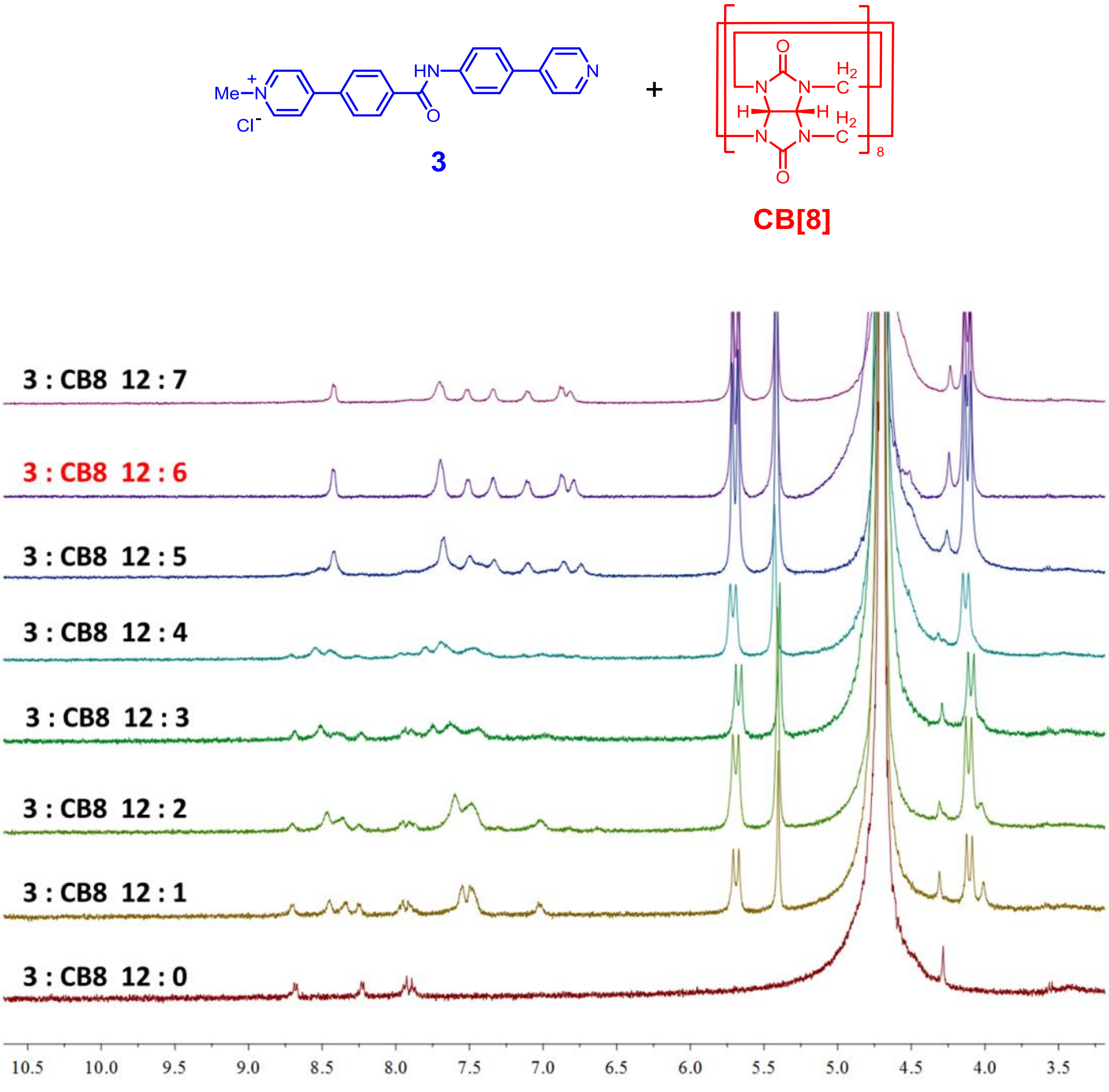

Supplementary Figure 3. ^{1}H NMR titration of **3** in D_2O with increasing amounts of CB[8] (400 MHz, 25 °C, [**3**] = 4.0 mM).

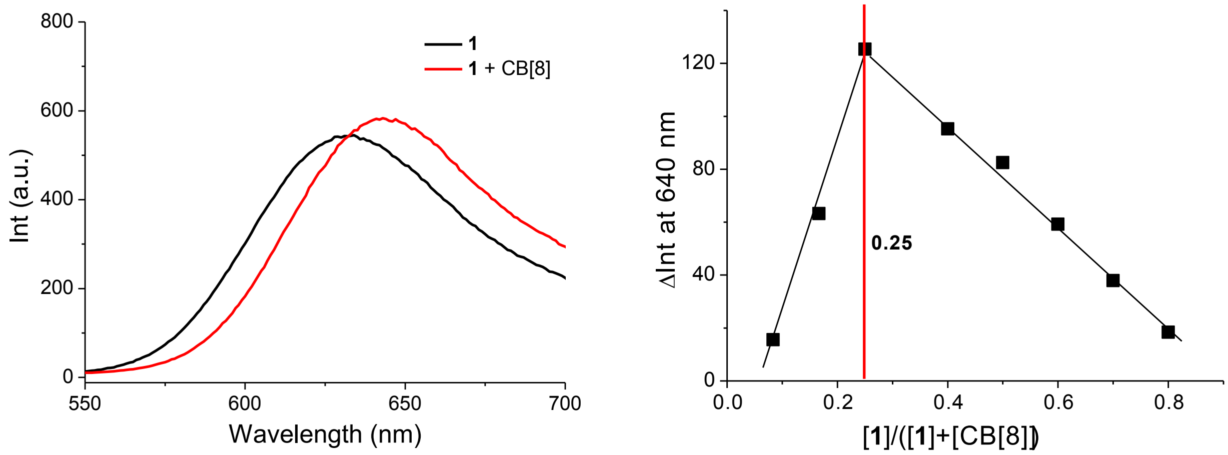

Supplementary Figure 4. Left: Fluorescence intensity of **1** with and without CB[8] in water ([**1**] = 0.05 mM, CB[8] = 0.15 mM). Right: Job Plots obtained by recording the fluorescence of **1** in water at 25 °C upon mixing with CB[8]. [**1**] + [CB[8]] = 0.2 mM, λ_{ex} = 480 nm, λ_{em} = 640 nm.

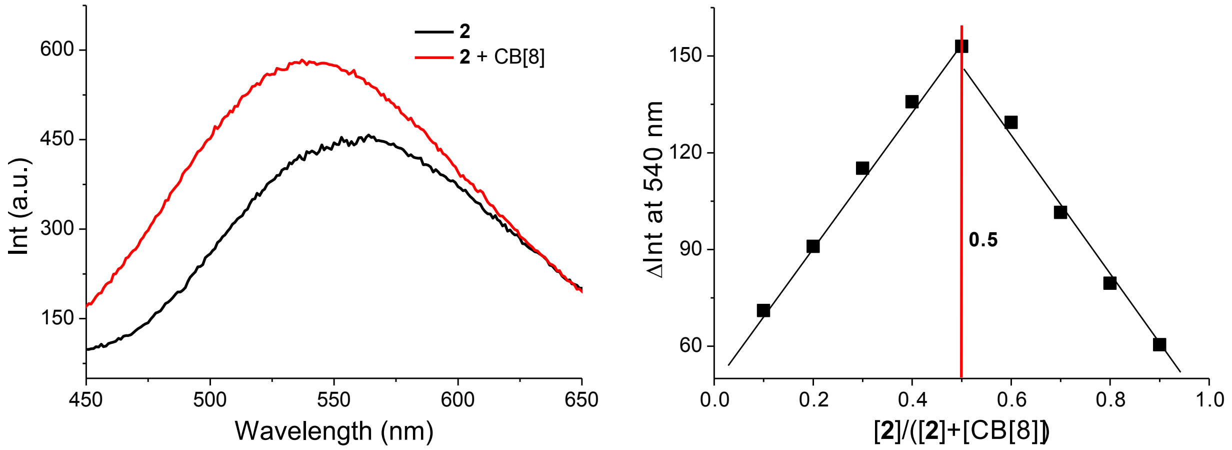

Supplementary Figure 5. Left: Fluorescence intensity of **2** with and without CB[8] in water ([**2**] = 0.15 mM, CB[8] = 0.15 mM). Right: Job plots obtained by recording the fluorescence of **2** in water at 25 °C upon mixing with CB[8]. [**2**] + [CB[8]] = 0.3 mM, λ_{ex} = 360 nm, λ_{em} = 540 nm.

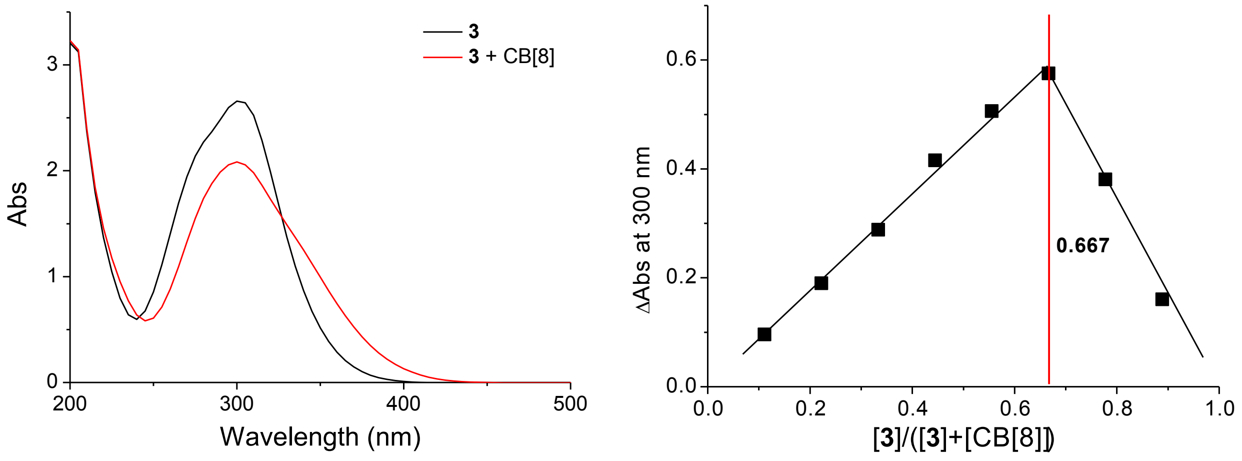

Supplementary Figure 6. Left: Absorbance of **3** with and without CB[8] in water ([**3**] = 0.15 mM, CB[8] = 0.075 mM). Right: Job plot obtained by recording the absorbance of **3** at 300 nm in water at 25 °C upon mixing with CB[8]. [**3**] + [CB[8]] = 0.225 mM.

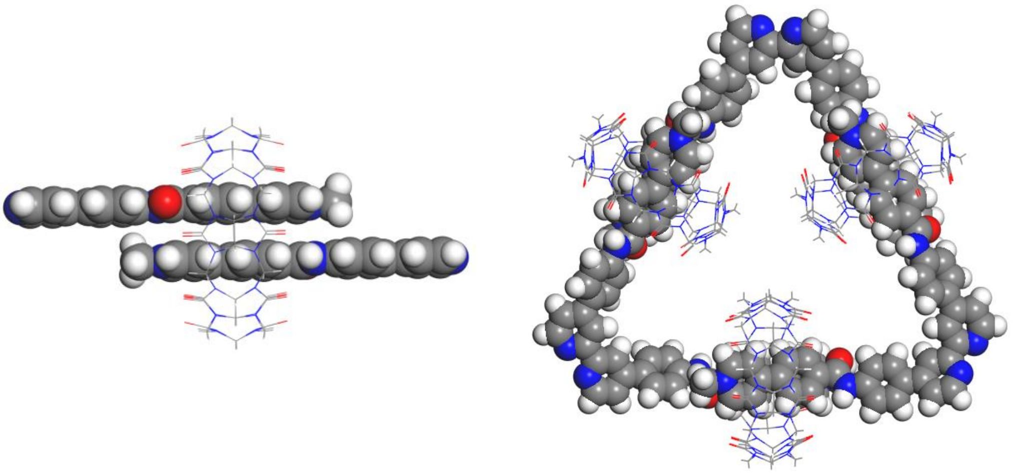

Supplementary Figure 7. Proposed self-assembled patterns. Left: Model of the 2:1 complex of **3** and CB[8] (**CP-a**). Right: Model of the macrocyclic 3+3 complex $\mathbf{2}_3$•CB[8]$_3$ (**CP-b**).

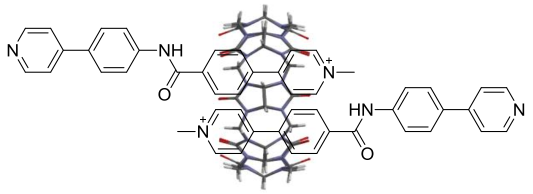

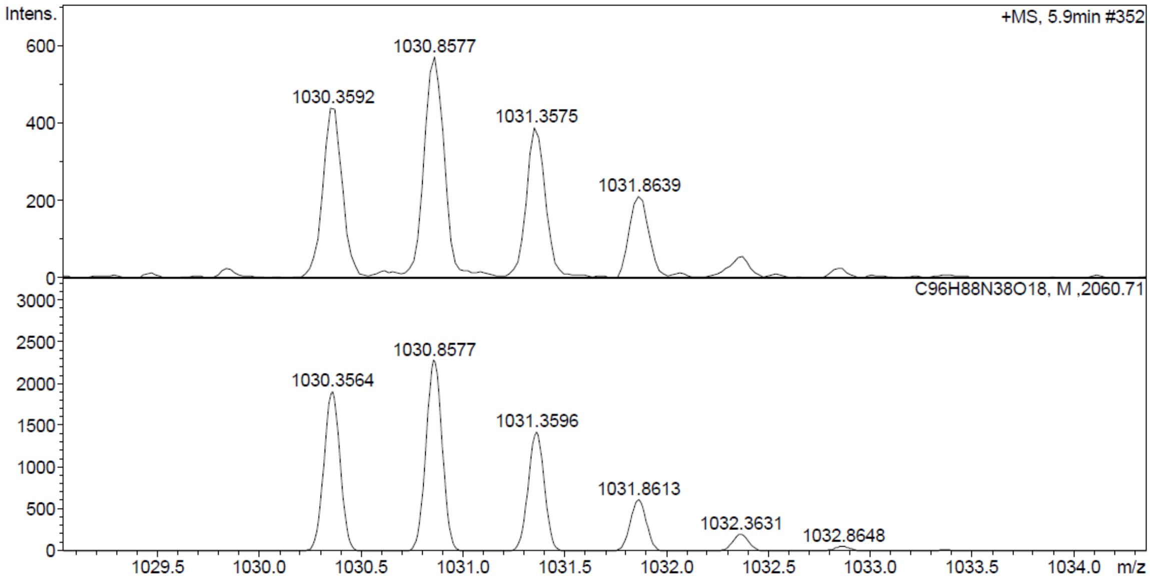

Supplementary Figure 8. ESI-mass spectrometry of the mixture solution of **3** and CB[8] (2:1) in water.

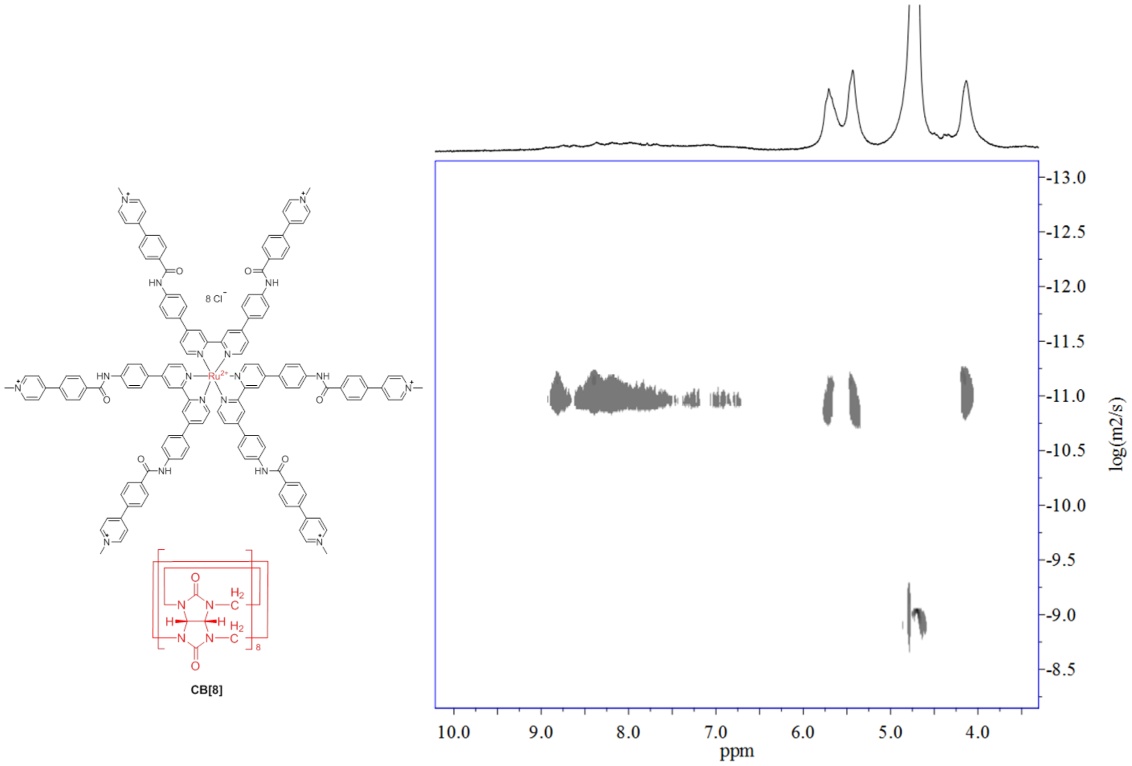

Supplementary Figure 9. DOSY ^{1}H NMR spectrum (400 MHz) of the solution of **1** (1.0 mM) and CB[8] (3.0 mM) in D_2O. The ordinate represents the log value of the diffusion constant.

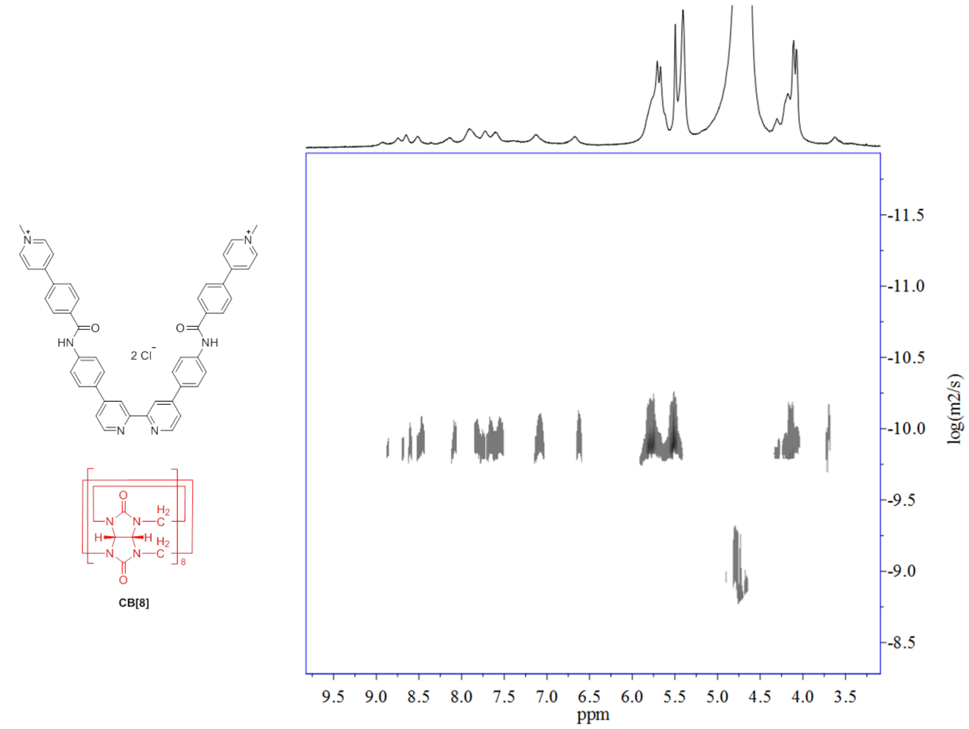

Supplementary Figure 10. DOSY ^{1}H NMR spectrum (400 MHz) of the solution of **2** (3.0 mM) and CB[8] (3.0 mM) in D_2O. The ordinate represents the log value of the diffusion constant.

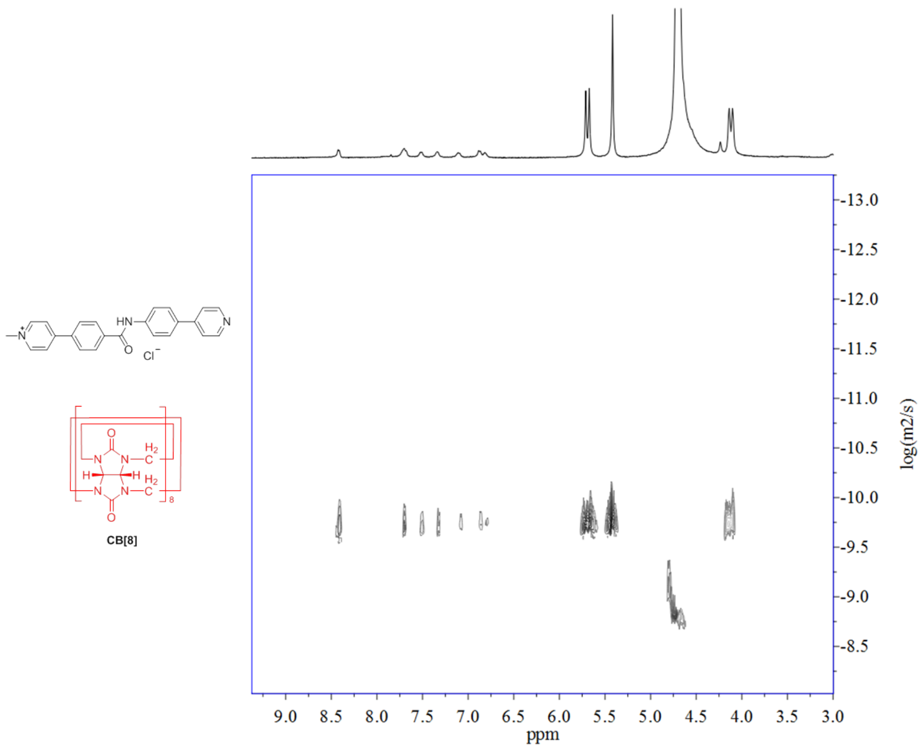

Supplementary Figure 11. DOSY ^{1}H NMR spectrum (400 MHz) of the solution of **3** (6.0 mM) and CB[8] (3.0 mM) in D_2O. The ordinate represents the log value of the diffusion constant.

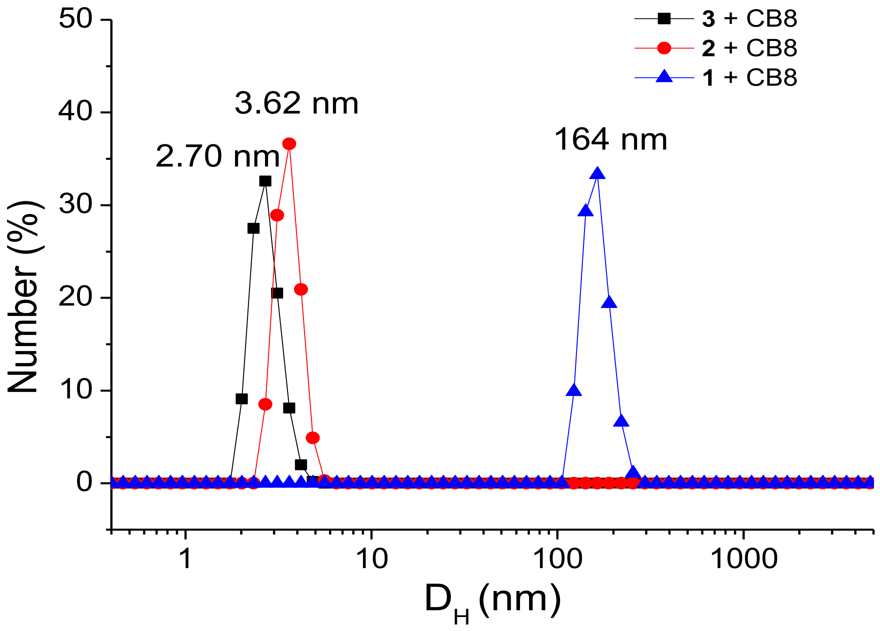

Supplementary Figure 12. DLS profiles of the mixtures of compounds **1-3** with CB[8] in water at 25 ℃. The data represent the hydrodynamic diameters (D_H). [**1**] = 2.0 mM, [**2**] = 6.0 mM, [**3**] = 12.0 mM, and CB[8] = 6.0 mM. The peak values of the solutions of compounds **1-3** and CB[8] were originally afforded by the instrument as 164±32, 3.62±0.87 and 2.70±0.57 nm, respectively.

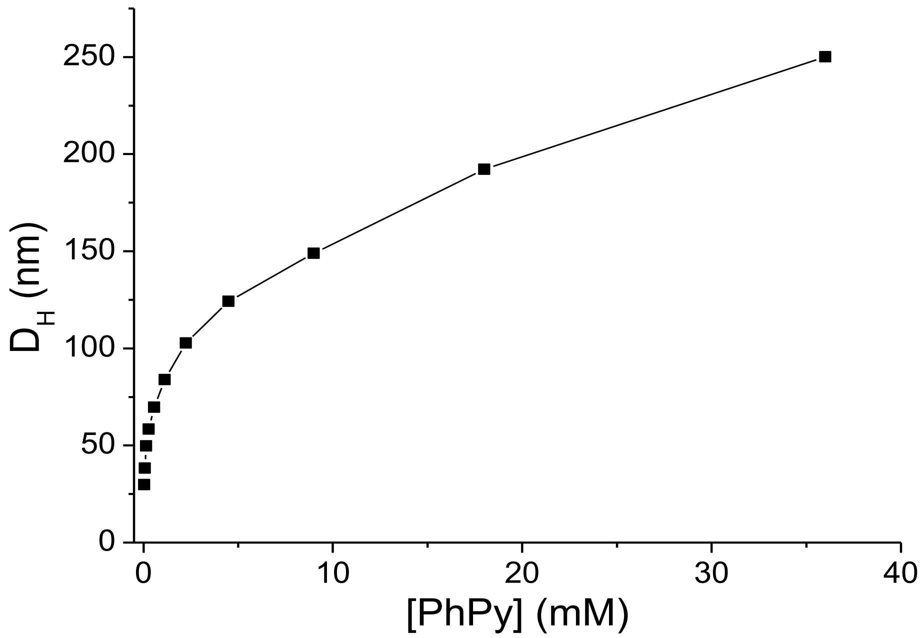

Supplementary Figure 13. The concentration dependence of D_{H} of the assemblies of compound **1** and CB[8] (1:3) in water at 25 ℃.

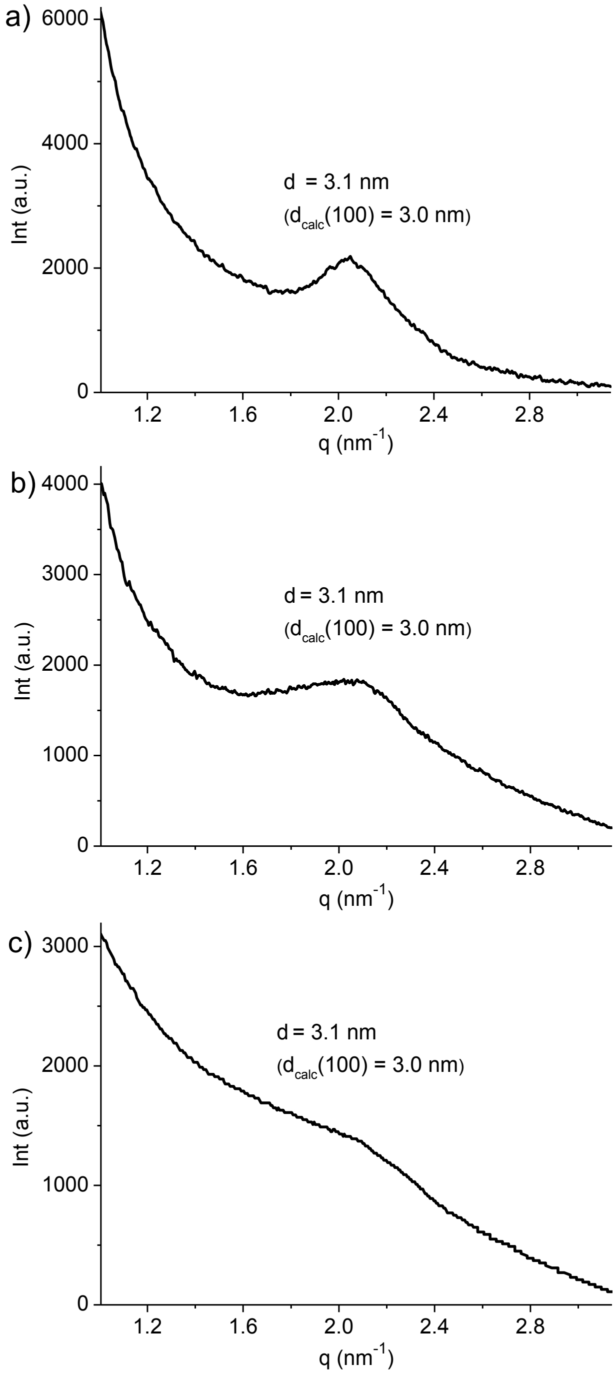

Supplementary Figure 14. Solution-phase synchrotron small-angle X-ray-scattering profiles of the solution of **SMOF-1** at different concentration in water. a.u.: arbitrary units. (a) [**1**] = 3.0 mM; (b) [**1**] = 1.5 mM; (c) [**1**] = 0.60 mM.

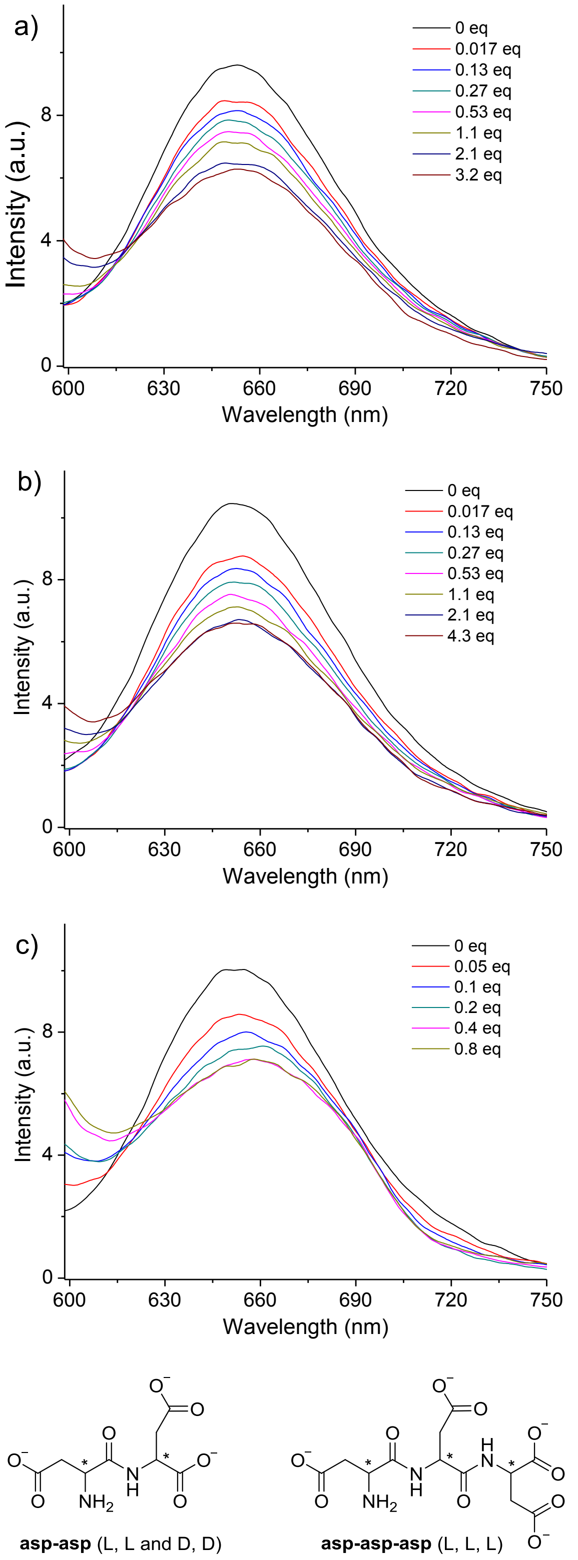

Supplementary Figure 15. Fluorescence spectra of **SMOF-1** ([**1**] = 0.02 mM) in water in the presence of incremental amount of anionic aspartic acid-derived (a and b) dipeptides **asp-asp** (L,L and D,D) and c) tripeptide **asp-asp-asp** (L,L,L). The concentration of the guests was relative to [**1**].

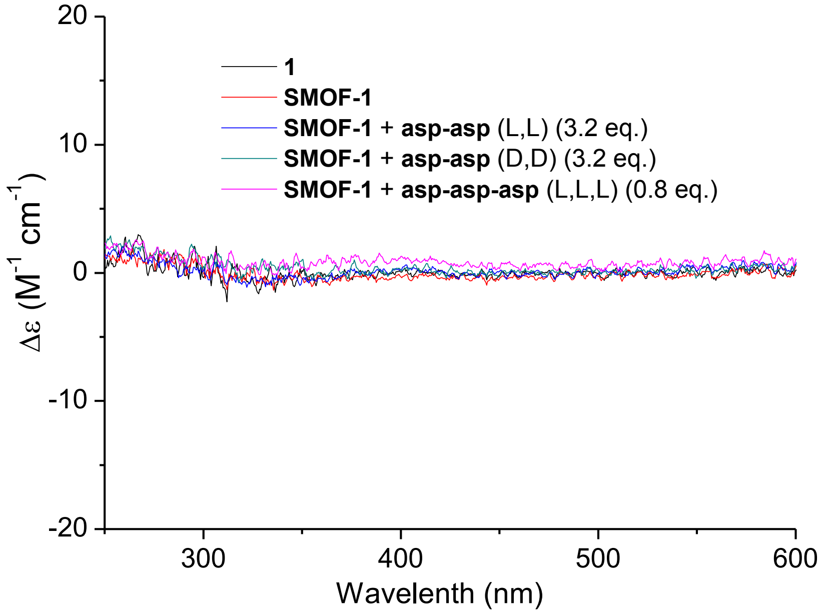

Supplementary Figure 16. Circular dichroism (CD) spectra of the aqueous solution of **1**, **SMOF-1** and the mixtures of **SMOF-1** and anionic aspartic acid-derived chiral dipeptides **asp-asp** and tripeptide **asp-asp-asp** (as sodium salt) at 25 °C ([**1**] = 0.02 mM). The concentration of the peptides was relative to [**1**].

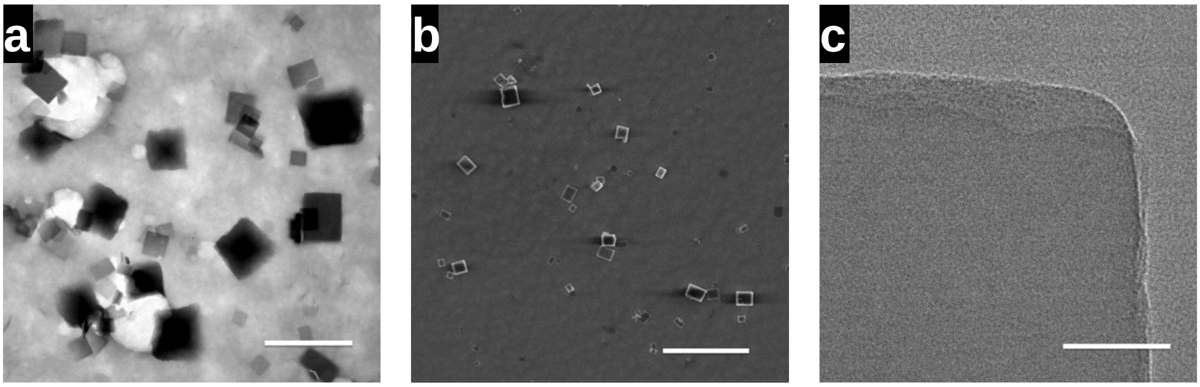

Supplementary Figure 17. (**a**) TEM images of **SMOF-1,** Scale bar = 2 μm. (**b**) SEM image of **SMOF-1,** Scale bar = 5 μm. (**c**) HR-TEM image of **SMOF-1**. Scale bar = 50 nm.

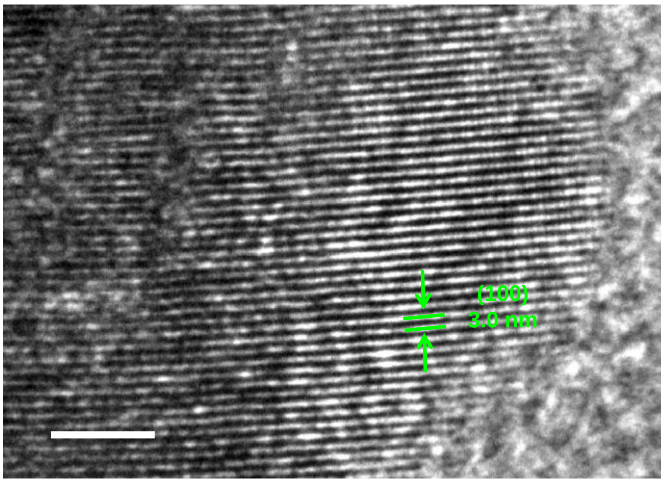

Supplementary Figure 18. High-resolution cryo-TEM image of **SMOF-1**, showing the lattice spacing. Scale bar = 20 nm.

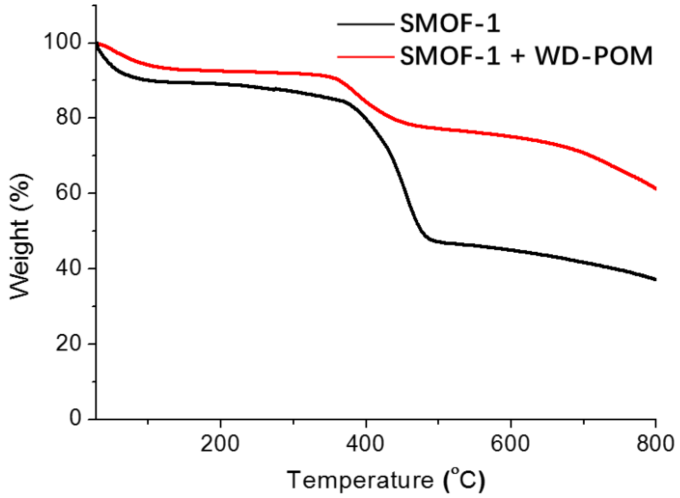

Supplementary Figure 19. TGA trace of SMOF-1 microcrystals and **WD-POM**-loaded **SMOF-1** ([**WD-POM**]/[**1**] = 0.78), which was pretreatment by dialysis in acid water (pH = 1.8) for three days before evaporation.

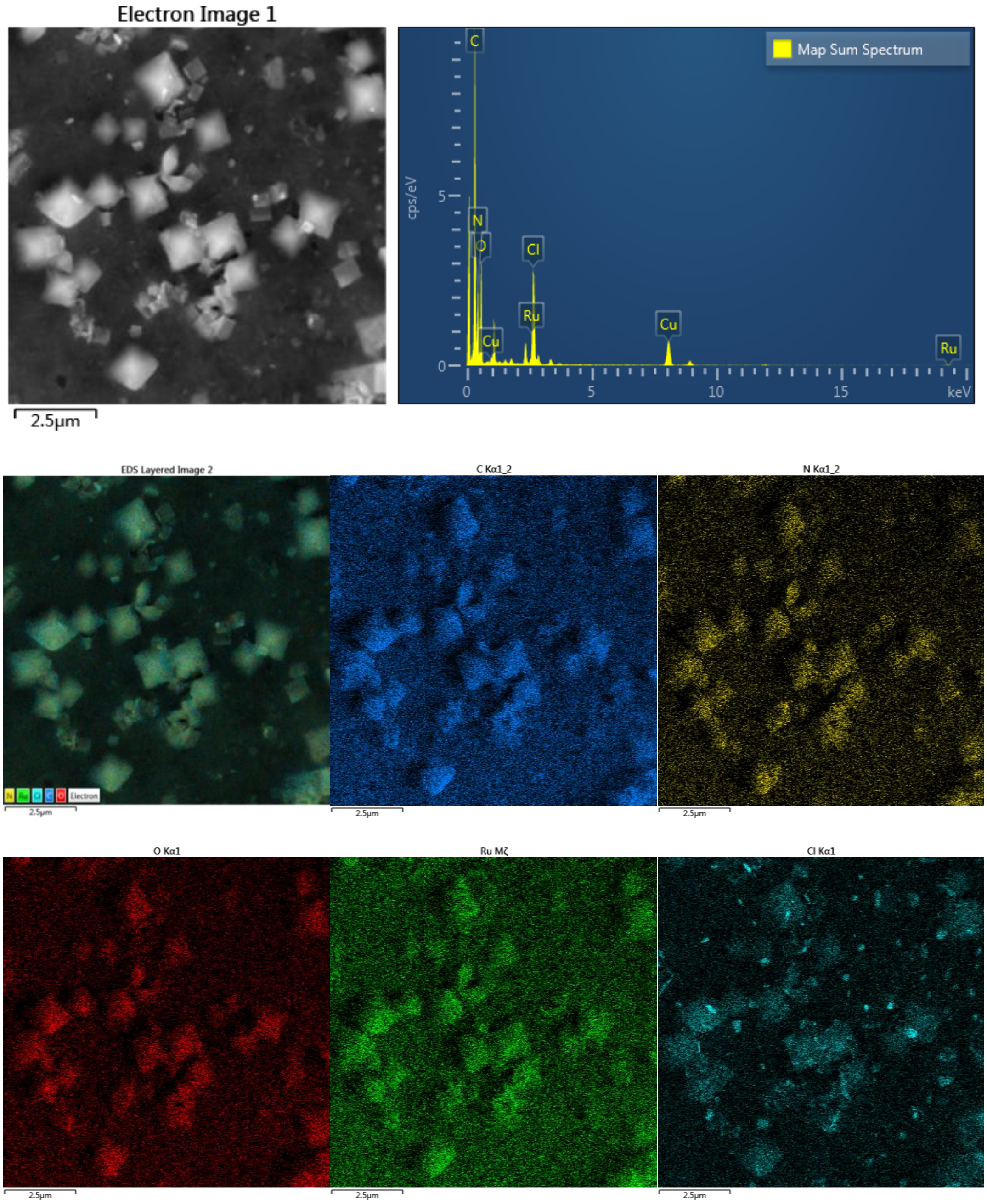

Supplementary Figure 20. Element distribution mapping images of **SMOF-1** microcrystals, showing the respective distribution of the C, N, O, Ru and Cl elements.

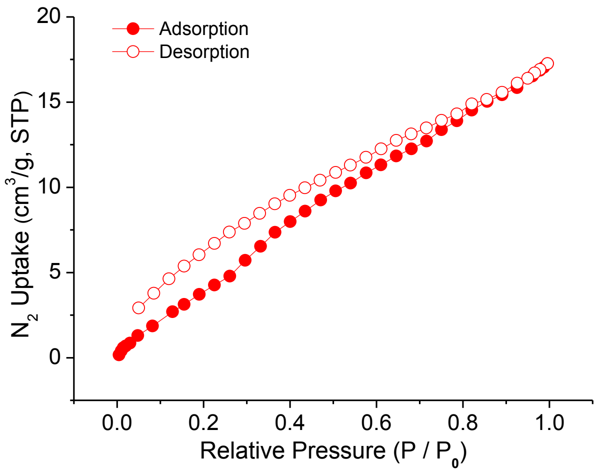

Supplementary Figure 21. Nitrogen gas adsorption and desorption isotherm curves of solid **SMOF-1** at 77 K.

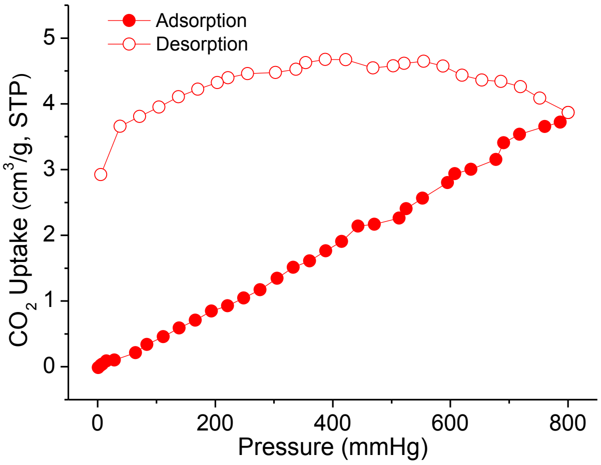

Supplementary Figure 22. Carbon dioxide gas adsorption isotherm curves of **SMOF-1** at 273 K.

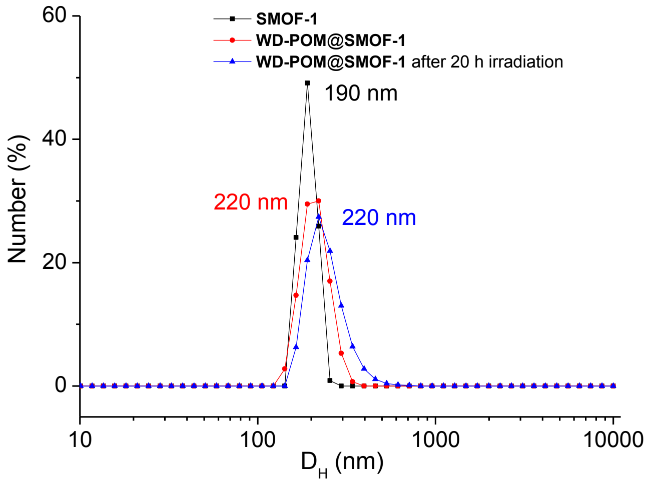

Supplementary Figure 23. DLS results of **SMOF-1** and **WD-POM@SMOF-1** before and after irradiation for 20 hours in water at 25 ℃. The data represent the hydrodynamic diameters (D_H). [**1**] = 3.0 mM, [**WD-POM**] = 0.2 mM.

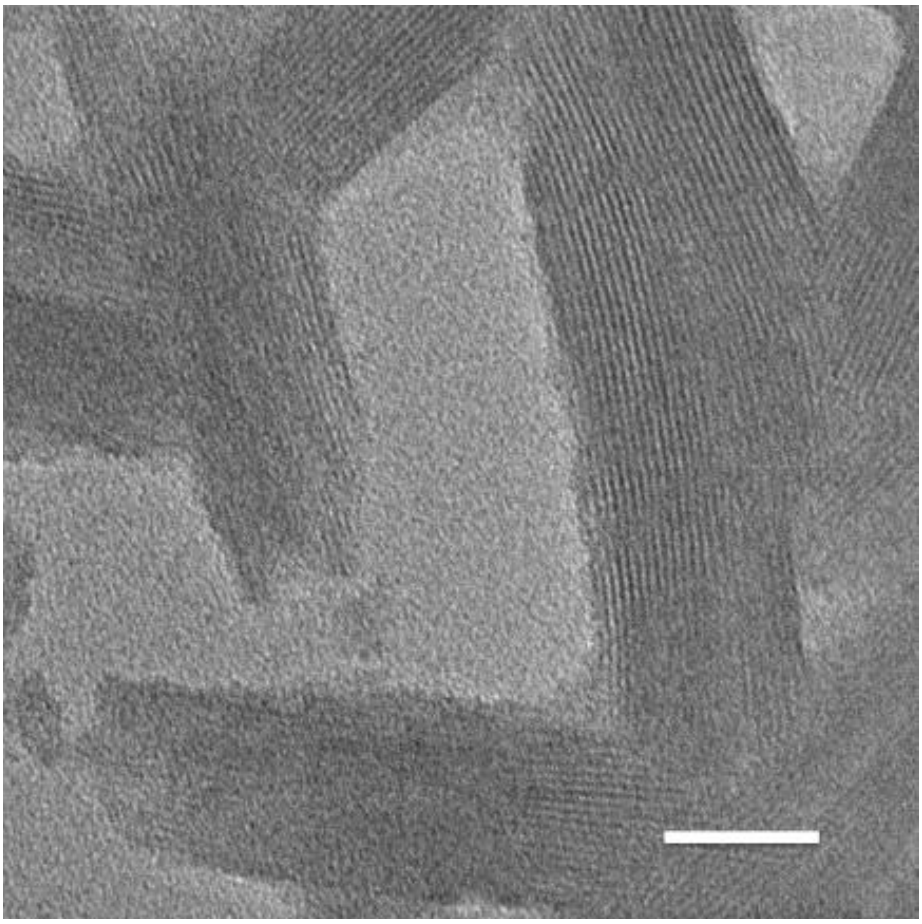

Supplementary Figure 24. High-resolution TEM of solid **WD-POM**-loaded **SMOF-1** viewed from the {110} orientation, showing the {100} lattice spacing (3.0 nm). Scale bar = 50 nm.

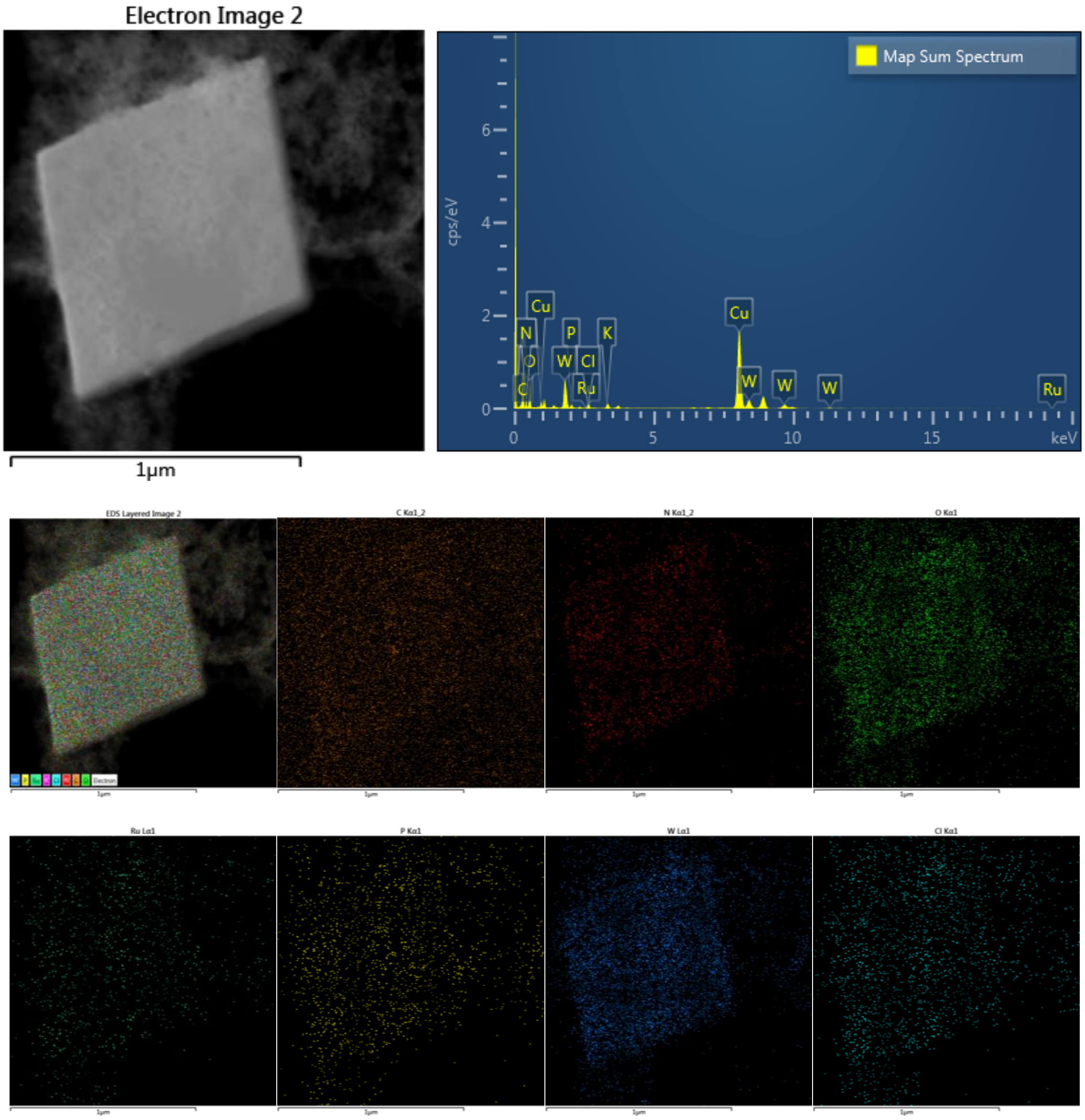

Supplementary Figure 25. Element distribution mapping images of the microcrystals of **WD-POM**-loaded **SMOF-1**, showing the respective distribution of the C, N, O, Ru, P, W and Cl elements.

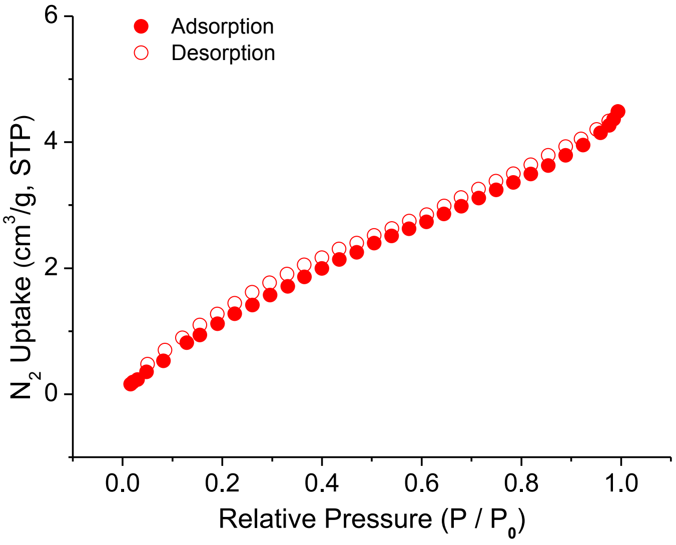

Supplementary Figure 26. Nitrogen gas adsorption and desorption isotherm curves of solid **WD-POM@SMOF-1** at 77 K.

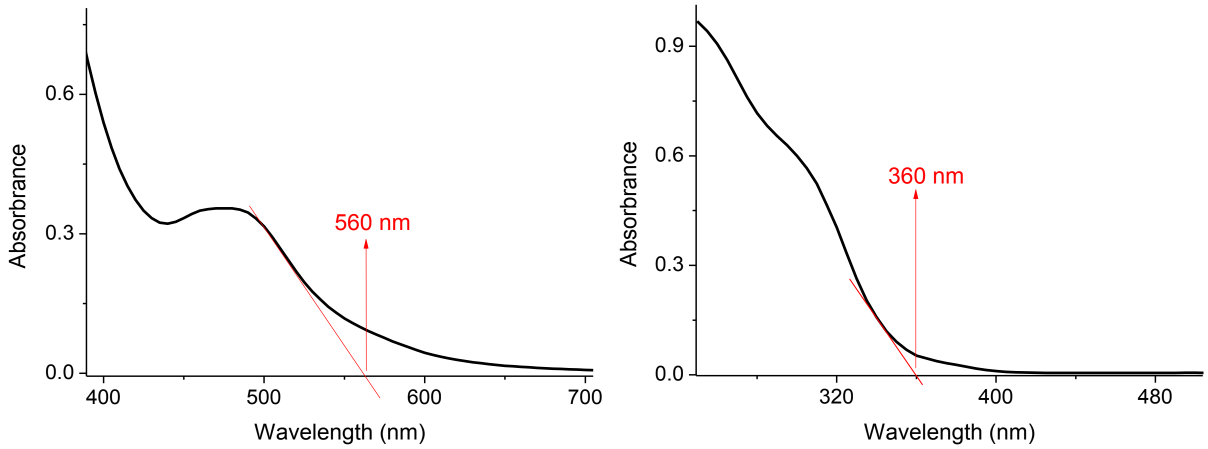

Supplementary Figure 27. UV-vis spectra of **SMOF-1** (left) and **WD-POM** (right) in the aqueous solution of Na_2SO_4 (0.2 M) and H_2SO_4 (0.2 M) (25 °C, pH = 1.8, [**1**] =0.020 mM, [**WD-POM**] = 0.02 mM).

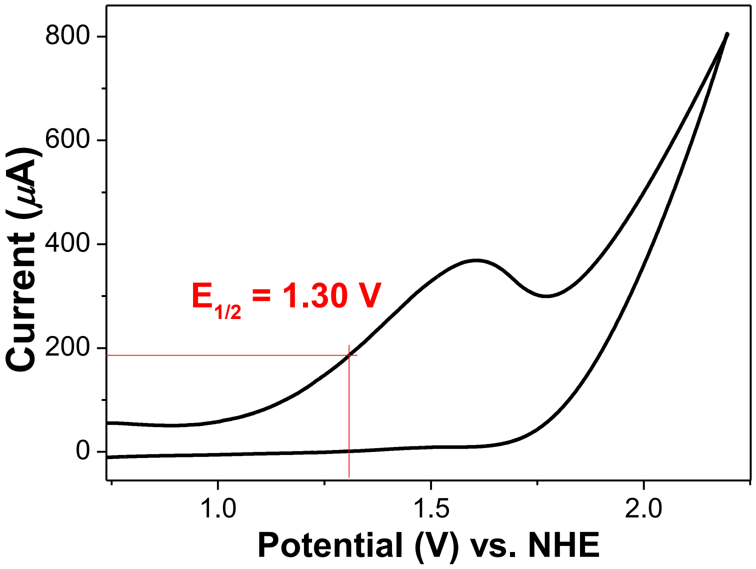

Supplementary Figure 28. Cyclic voltammetry of **WD-POM** (0.2 mM) in the aqueous solution of Na_2SO_4 (0.2 M) and H_2SO_4 (0.2 M) (pH = 1.8) with a scan rate of 100 mV/s. Working, reference, and counter electrodes are glassy carbon, Ag/AgCl, and Pt, respectively.

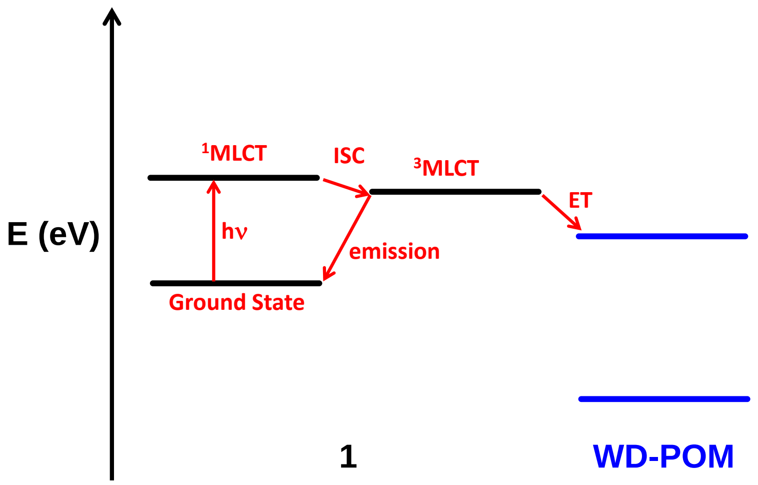

Supplementary Figure 29. Energy level diagram for complex **1** and **WD-POM**. Photon absorption generates the singlet excited-state (^{1}MLCT) of complex **1** which interconverts to the triplet state (^{3}MLCT) by inter-system crossing (ISC). The triplet state decay to the ground state is in competition with two other processes: emission or electron transfer (ET) to the LUMO of the **WD-POM** which enables the hydronium reduction.

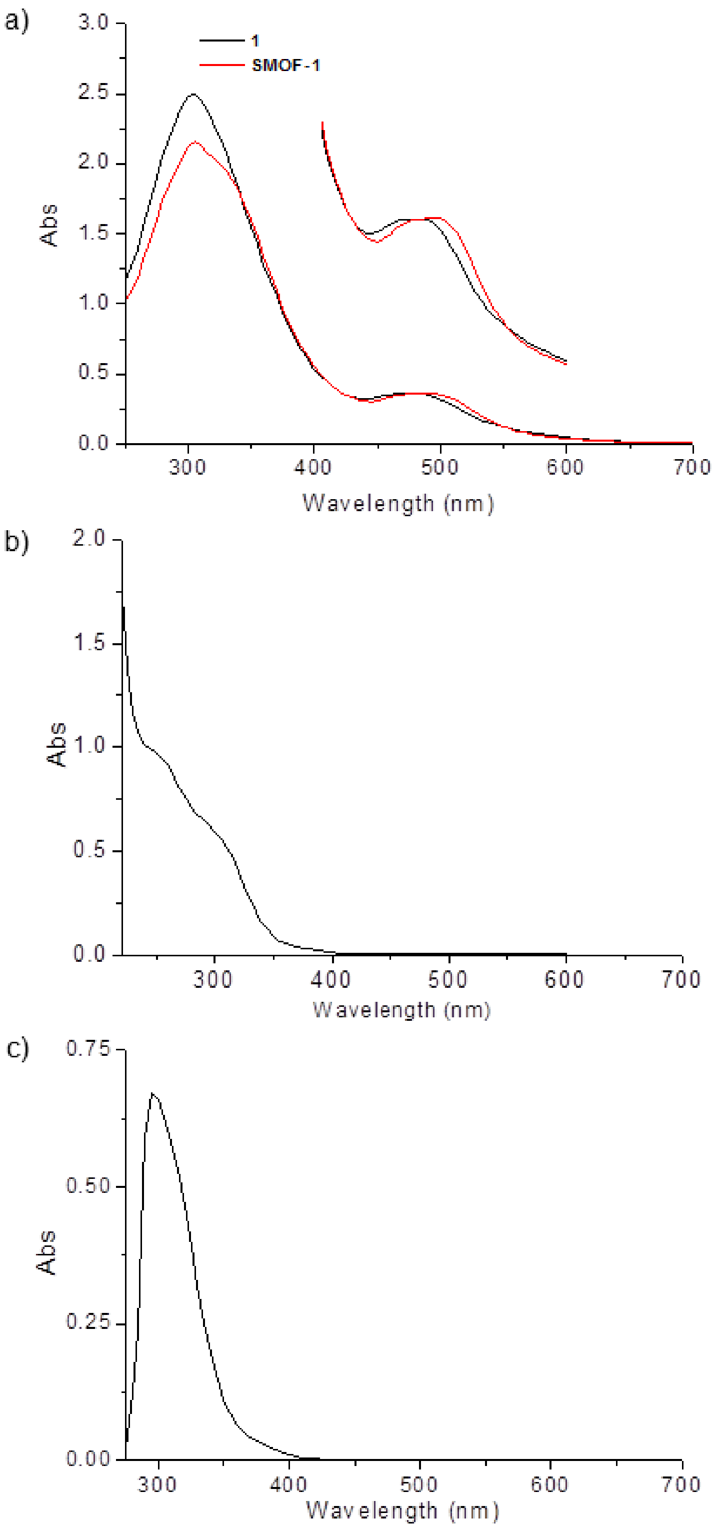

Supplementary Figure 30. a) UV-vis spectrum of complex **1** (0.02 mM) and **SMOF-1** ([**1**] = 0.02 mM) in water at 25 °C. The maximum absorbance in the visible range has a red shift from 470 nm (**1**) to 500 nm (**SMOF-1**). b) UV-vis spectrum of **WD-POM** (0.02 mM) in water/MeOH (10:1, v/v, pH = 1.8 adjusted with HCl). c) UV-vis spectrum of **WD-POM** (0.02 mM) in DMF/MeCN/ triethanolamine/water (14:6:1:2 v/v, pH = 2.4 adjusted with HCl) at 25 °C.

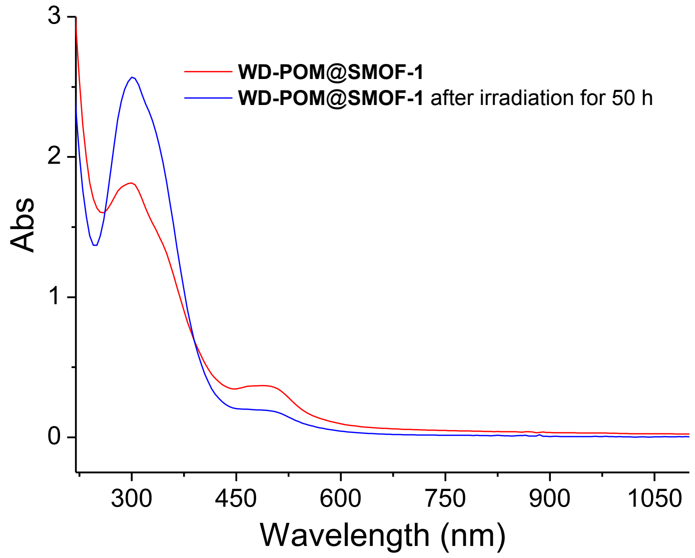

Supplementary Figure 31. UV-vis spectra of **SMOF-1** and **WD-POM@SMOF-1** before and after irradiation for 50 hours in water (25 °C, [**1**] = 0.020 mM, [**WD-POM**] = 0.0013 mM).

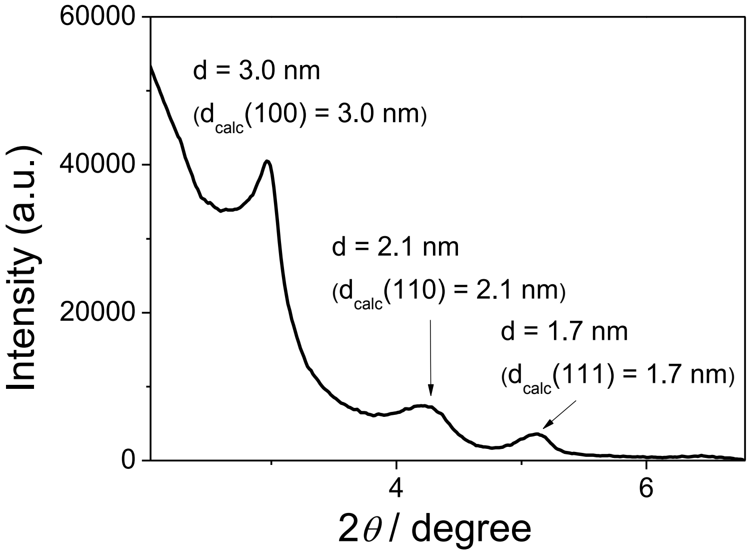

Supplementary Figure 32. Solid-phase XRD profile of **WD-POM@SMOF-1** after irradiation for 14 hours. The sample was obtained by slow evaporation of the aqueous solution. a.u.: arbitrary unit, [**1**] = 3.0 mM, [**WD-POM**] = 0.2 mM.

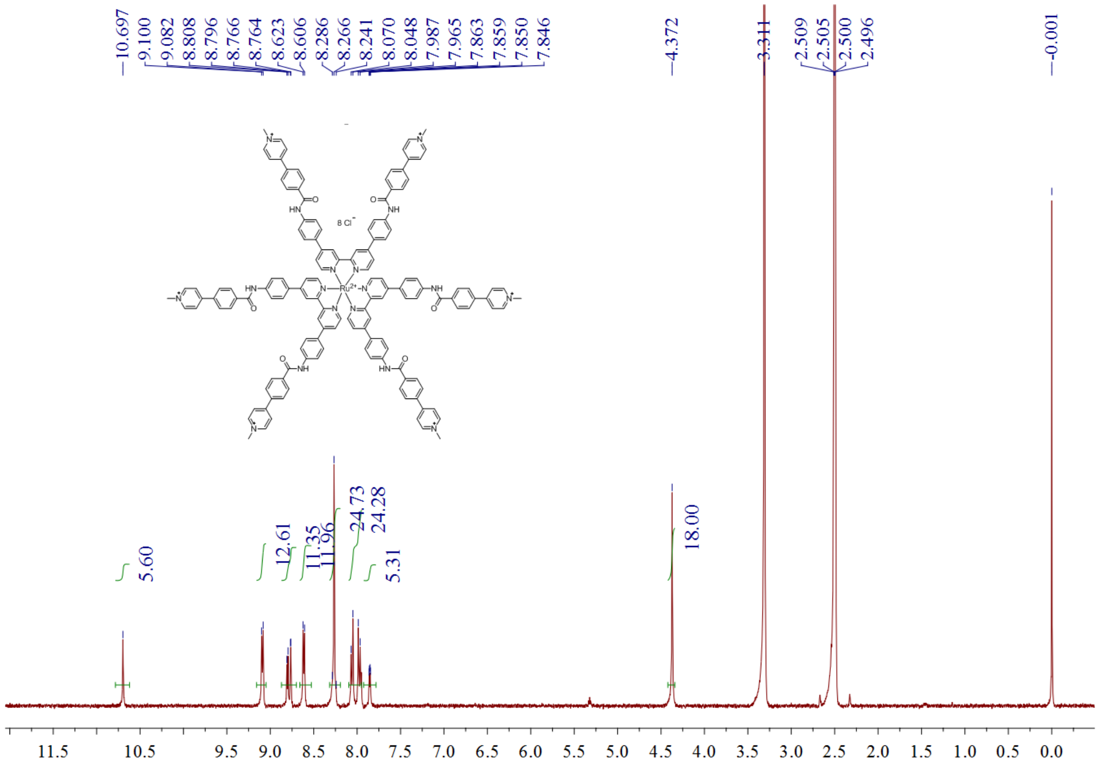

Supplementary Figure 33. ^{1}H NMR spectrum (400 MHz) of compound **1** in DMSO-d_6 (2 mM).

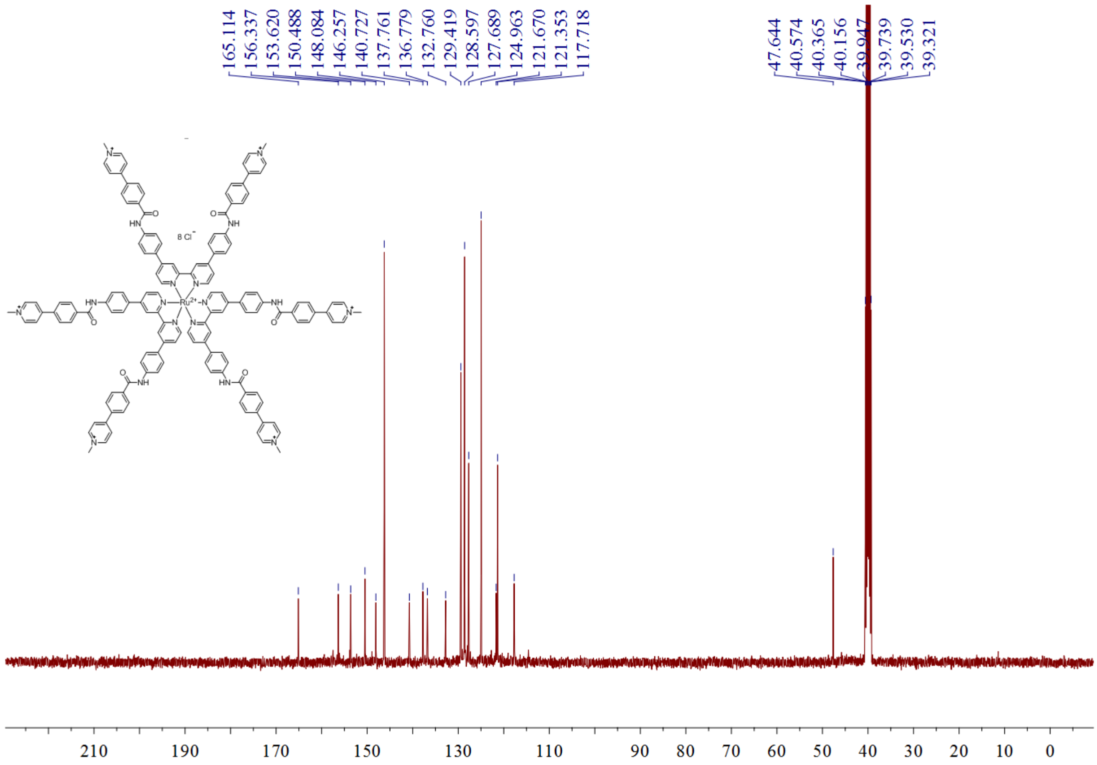

Supplementary Figure 34. ^{13}C NMR spectrum (100 MHz) of compound **1** in DMSO-d_6 (6 mM).

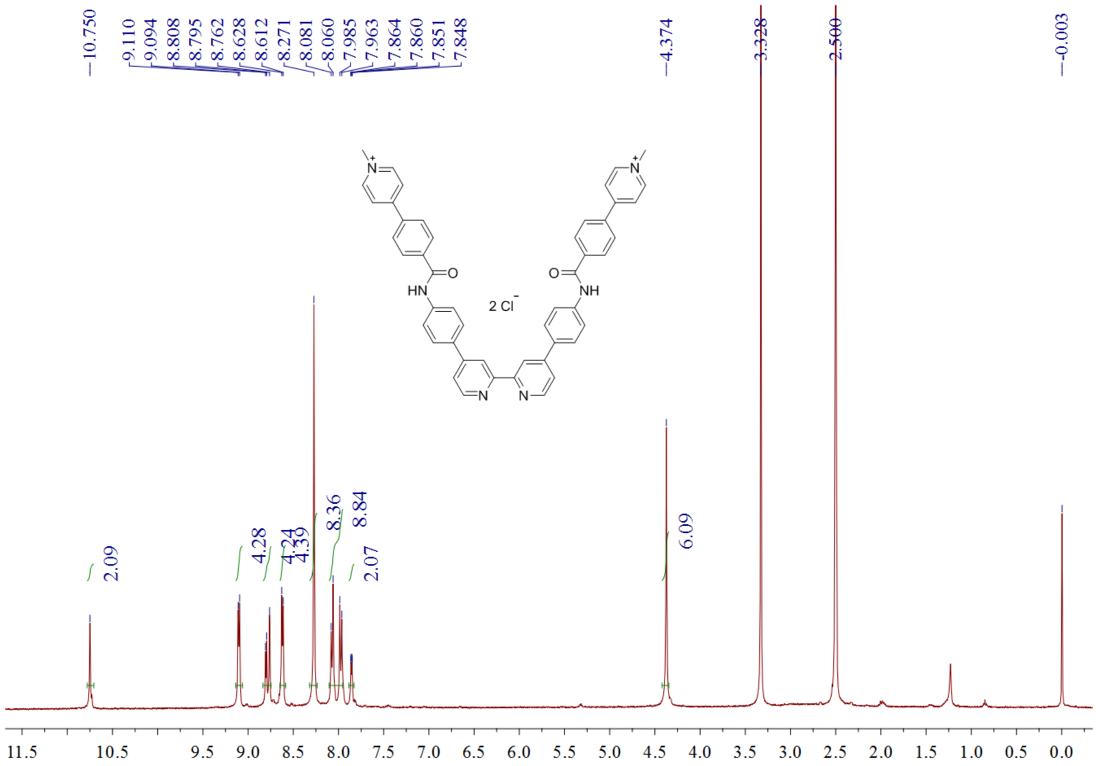

Supplementary Figure 35. ^{1}H NMR spectrum (400 MHz) of compound **2** in DMSO-d_6 (3 mM).

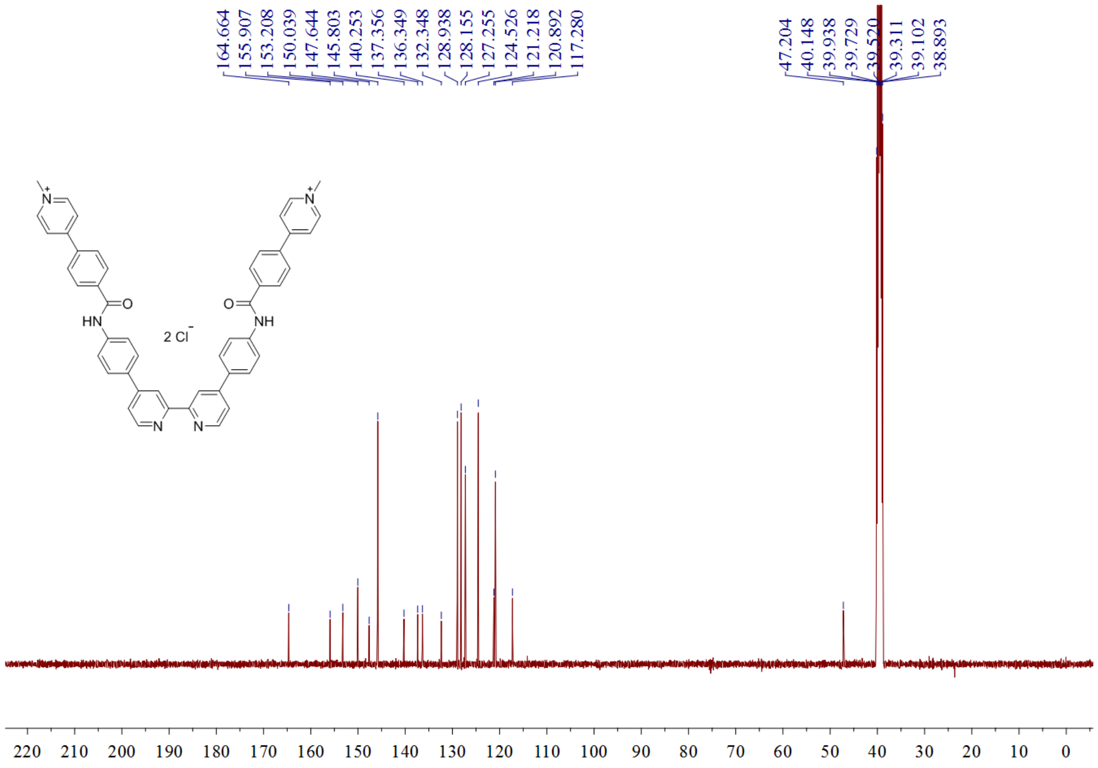

Supplementary Figure 36. ^{13}C NMR spectrum (100 MHz) of compound **2** in DMSO-d_6 (10 mM).

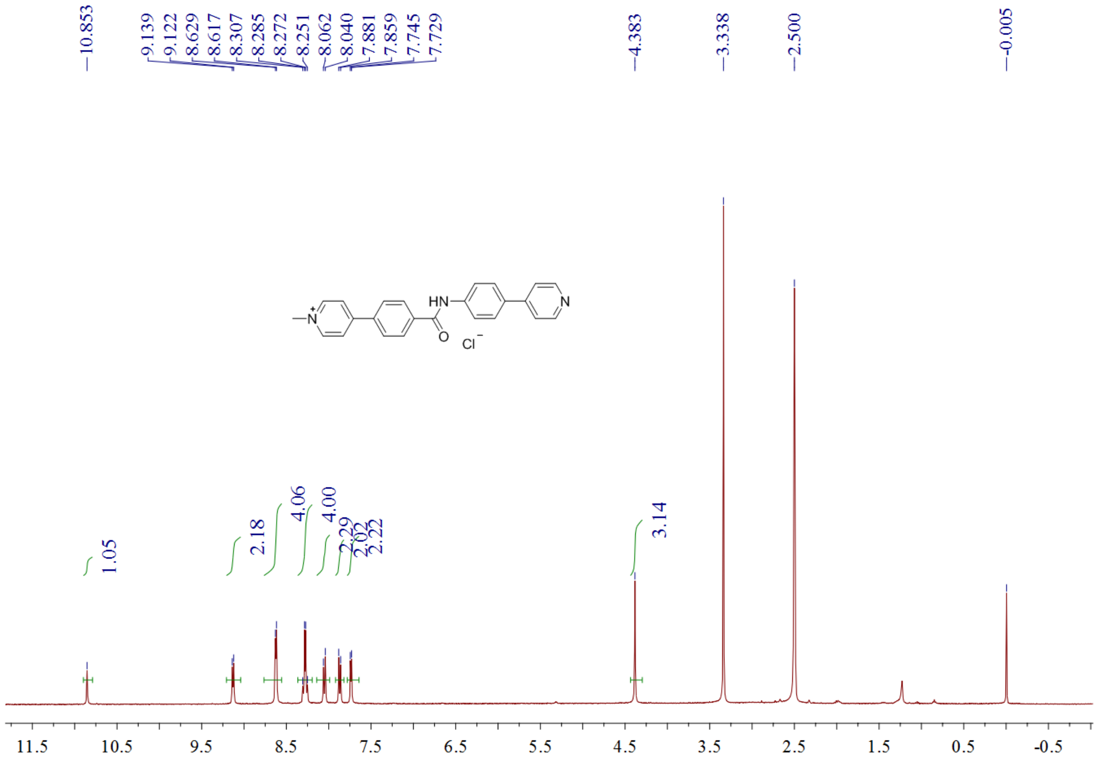

Supplementary Figure 37. ^{1}H NMR spectrum (400 MHz) of compound **3** in DMSO-d_6 (6 mM).

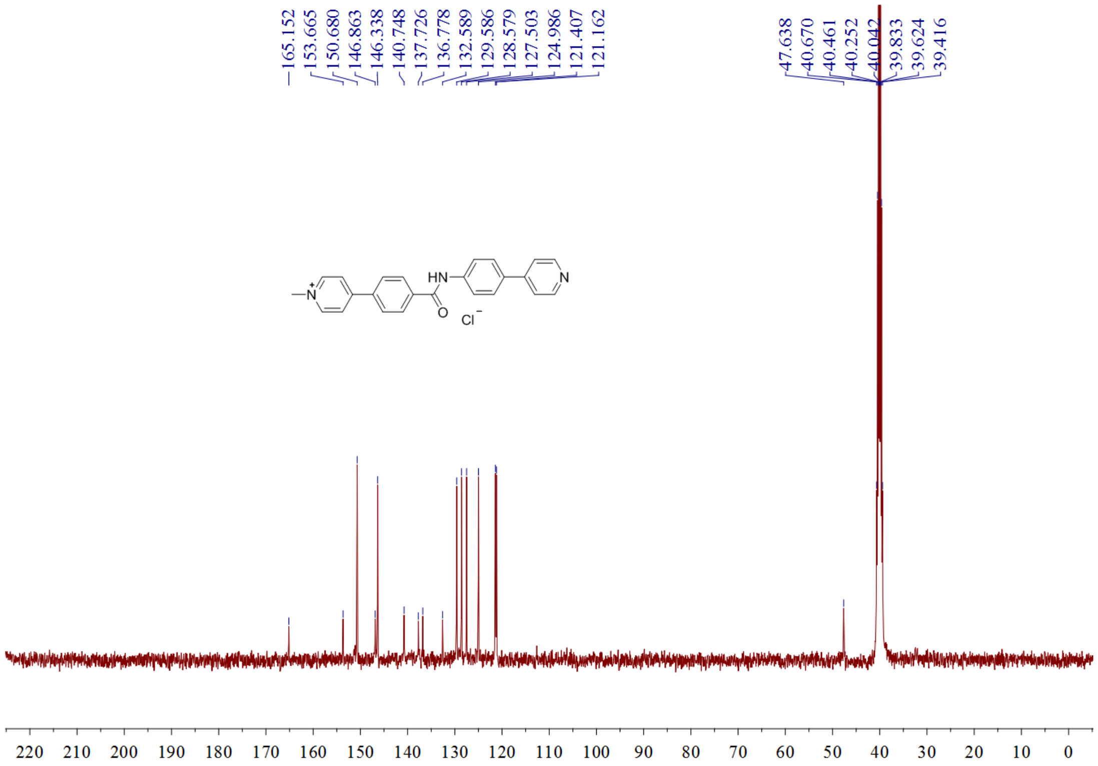

Supplementary Figure 38. ^{13}C NMR spectrum (100 MHz) of compound **2** in DMSO-d_6 (12 mM).

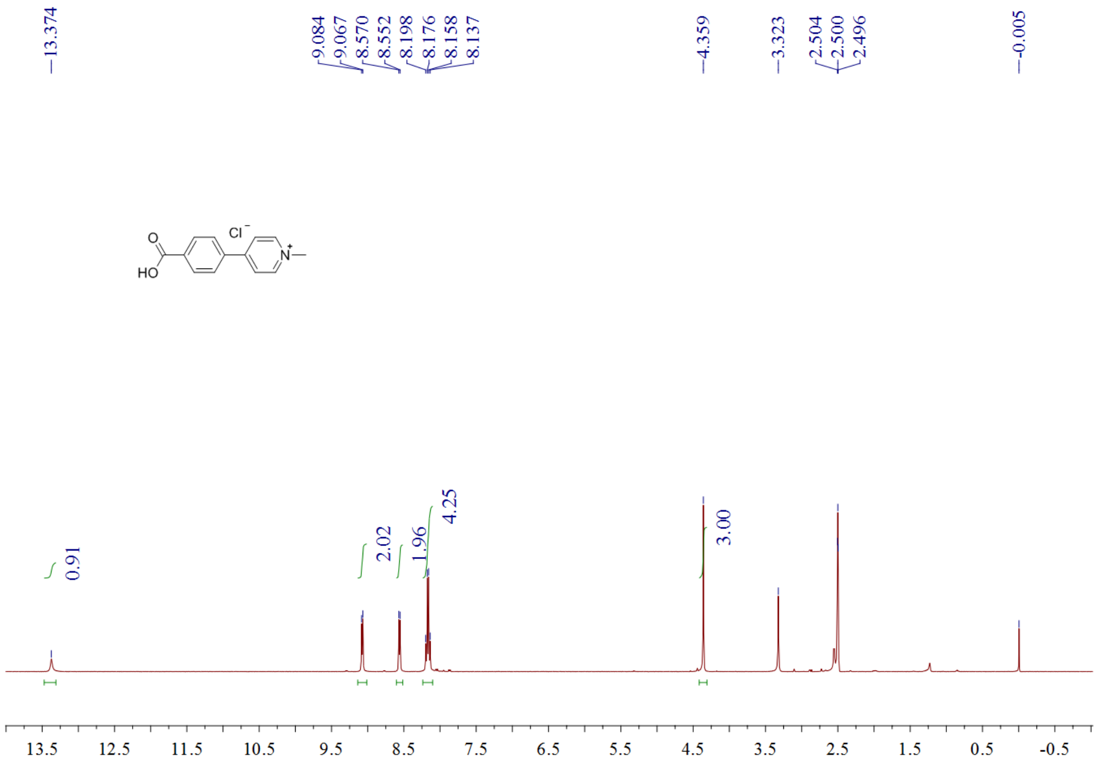

Supplementary Figure 39. ^{1}H NMR spectrum (400 MHz) of compound **5** in DMSO-d_6 (5 mM).

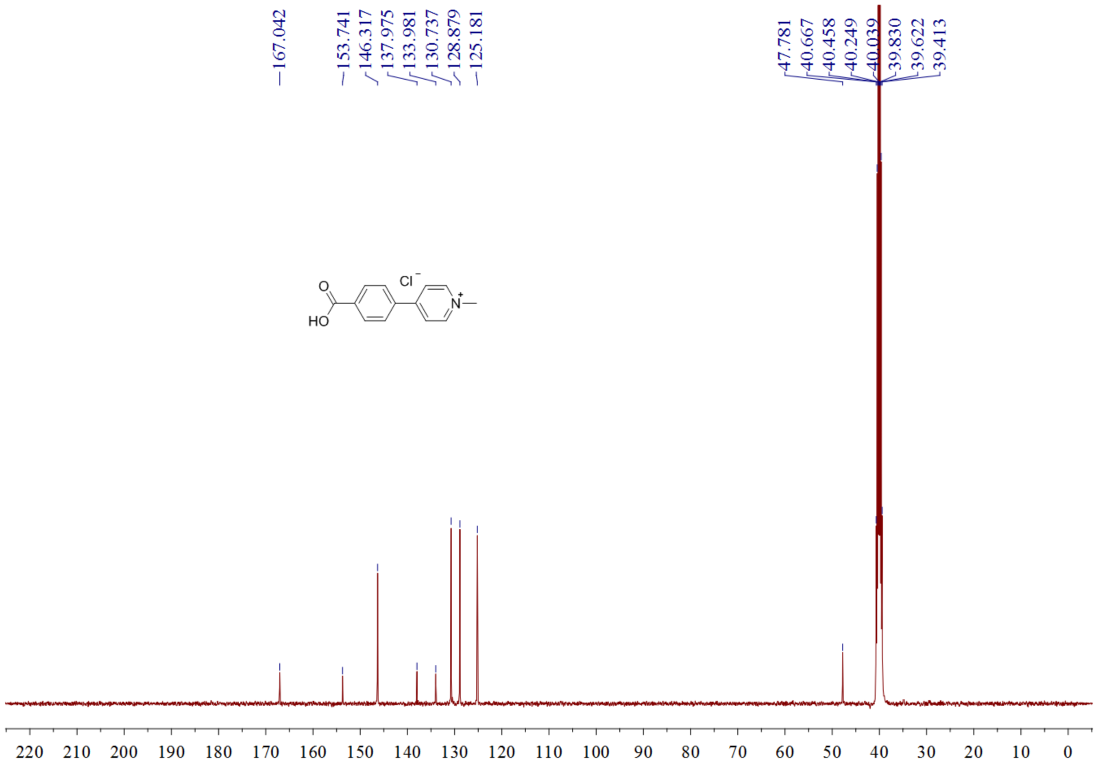

Supplementary Figure 40. ^{13}C NMR spectrum (100 MHz) of compound **5** in DMSO-d_6 (10 mM).

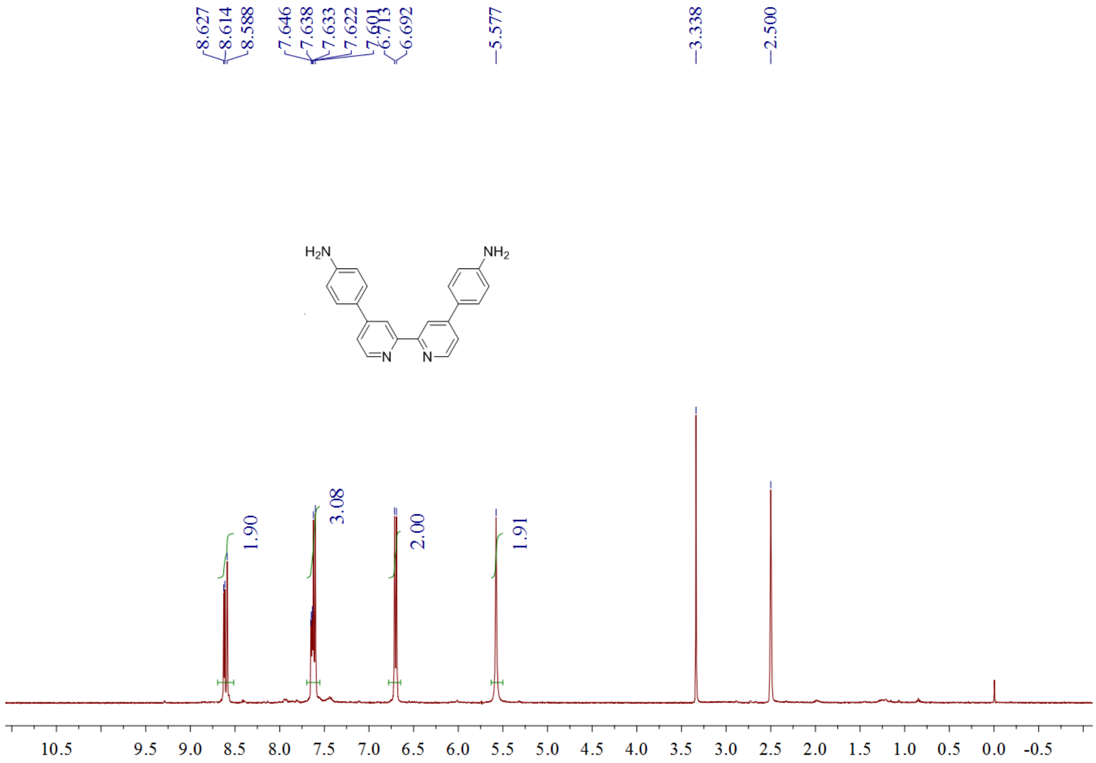

Supplementary Figure 41. ^{1}H NMR spectrum (400 MHz) of compound **9** in DMSO-d_6 (5 mM).

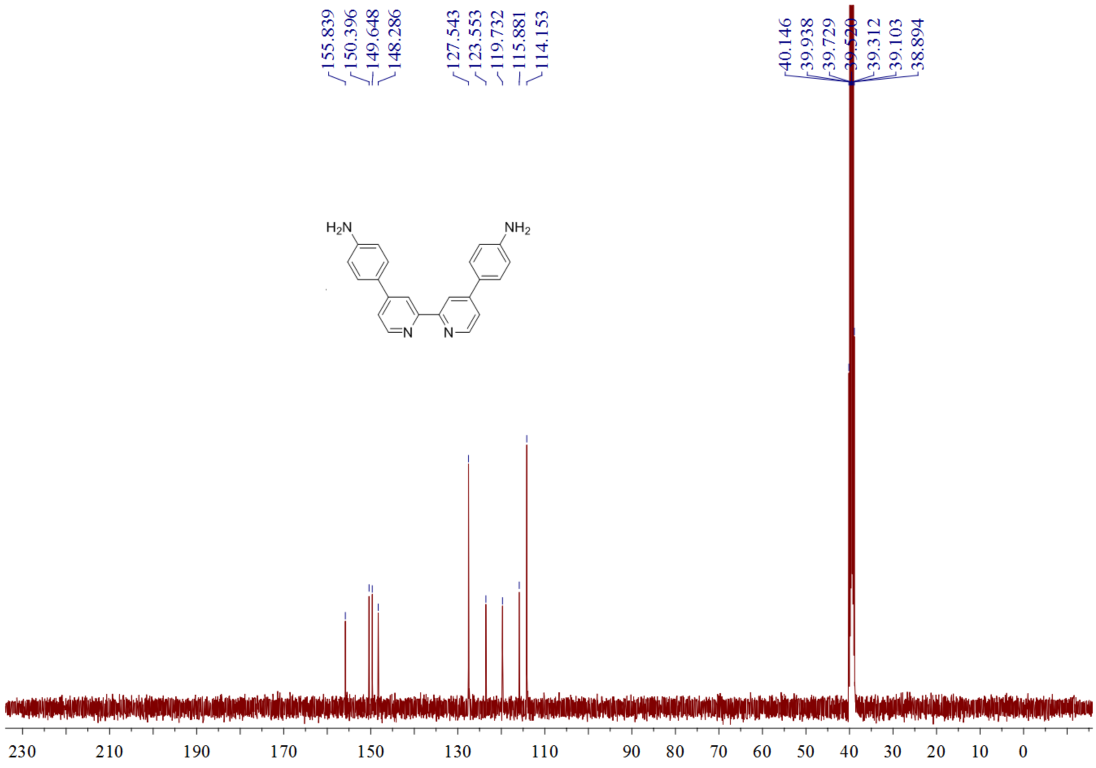

Supplementary Figure 42. ^{13}C NMR spectrum (100 MHz) of compound **9** in DMSO-d_6 (10 mM).

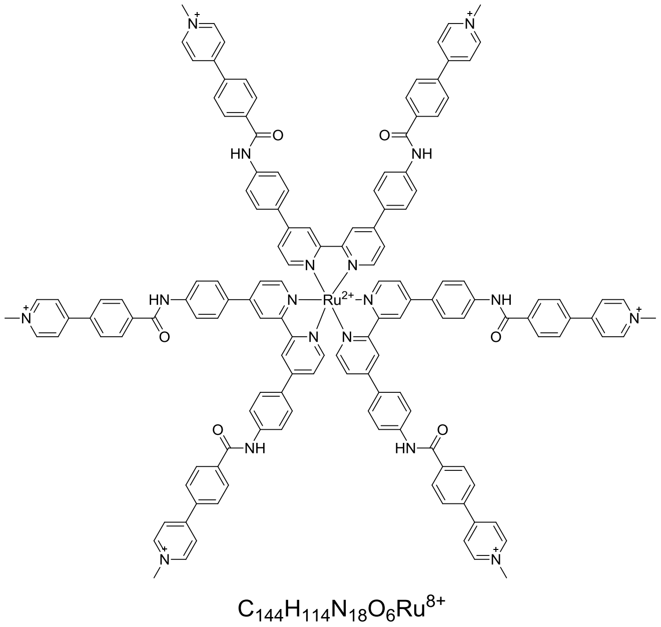

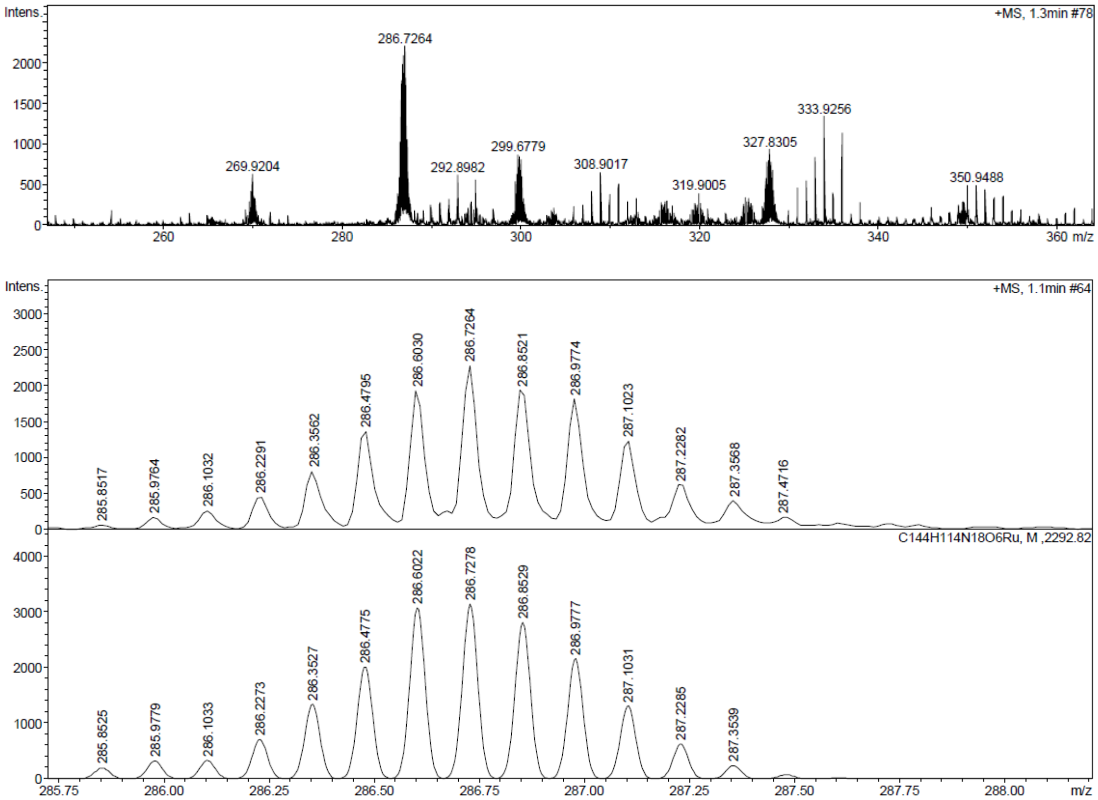

Supplementary Figure 43. ESI-mass spectrometry of compound **1**.

Supplementary Table 1. Homogeneous photocatalytic hydrogen production by **WD-POM**-loaded **SMOF-1** system and the control experiments in aqueous solutions (1 mL) with methanol as the sacrificial electron donor.

Entry	Photosensitizer	Catalyst	[**1**] (mM)	[**WD-POM**] (mM)	MeOH (mL)	pH	Irradiation time (h)	TON[a]
1 (1st run)	**SMOF-1**	**WD-POM**	0.3	0.02	0.22	1.8	10	76
2 (2nd run)	**SMOF-1**	**WD-POM**	0.3	0.02	0.22	1.8	10	72
3 (3rd run)	**SMOF-1**	**WD-POM**	0.3	0.02	0.22	1.8	10	70
4 (4th run)	**SMOF-1**	**WD-POM**	0.3	0.02	0.22	1.8	10	65
5 (5th run)	**SMOF-1**	**WD-POM**	0.3	0.02	0.22	1.8	10	59
6 (6th run)	**SMOF-1**	**WD-POM**	0.3	0.02	0.22	1.8	10	45
7	**SMOF-1**	**WD-POM**	0.3	0.02	0.22	4.2	10	7
8	**SMOF-1**	**WD-POM**	0.3	0.02	0.22	7	10	2
Control 1	Compound **1**	**WD-POM**	0.3	0.02	0.22	1.8	10	1
Control 2	**SMOF-1**	no	0.3	0	0.22	1.8	10	0

[a]TON is defined as n(1/2H_2)/n(POM).

Supplementary Table 2. Heterogeneous photocatalytic hydrogen production by **WD-POM-loaded SMOF-1** microcrystals in MeCN and DMF (3:7) with methanol or triethanolamine (TEOA) as sacrificial electron donor.

Entry	Photosensitizer	Catalyst	[**1**] (mM)	[**WD-POM**] (mM)	MeOH (mL)	TEOA (mL)	pH	Irradiation Time (h)	TON
1	**SMOF-1**	**WD-POM**	0.3	0.020	0.25	0	2.4	12	48
2	**SMOF-1**	**WD-POM**	0.3	0.600	0	0.10	2.4	12	20
3	**SMOF-1**	**WD-POM**	0.3	0.200	0	0.10	2.4	12	21
4	**SMOF-1**	**WD-POM**	0.3	0.060	0	0.10	2.4	12	23
5	**SMOF-1**	**WD-POM**	0.3	0.020	0	0.10	2.4	12	115
6	**SMOF-1**	**WD-POM**	0.3	0.006	0	0.10	2.4	12	335
7	**SMOF-1**	**WD-POM**	0.3	0.002	0	0.10	2.4	12	1561
8	**SMOF-1**	**WD-POM**	0.3	0.002	0	0.10	2.4	14	1820

Supplementary Table 3. Estimated molecular orbital energy of **1** and **WD-POM**[1]

Compound	E_g^o (eV)[a]	$E_{1/2}^{re}$ (V)[b]	$E_{1/2}^{ox}$ (V)[b]	HOMO (eV)[c]	LUMO (eV)[c]
**1**	2.21[d]	-	1.30[f]	–5.80	–3.59
**WD-POM**	3.44[d]	0.28[e]	-	–8.22	–4.78

[a] Optical band gap E_g^o=1240 / λ_{onset}^{abs}

[b] Potentials versus normal hydrogen electrode (NHE).

[c] HOMO and LUMO energies were calculated with reference to NHE (4.50 eV) LUMO = -(4.50 + $E_{1/2}^{re}$); HOMO = -(4.50 + $E_{1/2}^{ox}$); HOMO = LUMO – E_g^o.

[d] The optical band gap estimated from the tangents of the absorption edges of their UV/Vis spectra (Supplementary Fig. 27).

[e] The reduction potential of **WD-POM** was obtained from the reported cyclic voltammetry[2].

[f] The oxidation potential was obtained from the cyclic voltammetry (Supplementary Figure 28).

Supplementary Methods

Compounds **4**, **6**, **7**, and **8** were purchased from Sigma-Aldrich Chemical Co. $K_6P_2W_{18}O_{64}·14H_2O$ was synthesized according to literature[3]. All reagents were obtained from commercial suppliers and used without further purification unless otherwise noted.

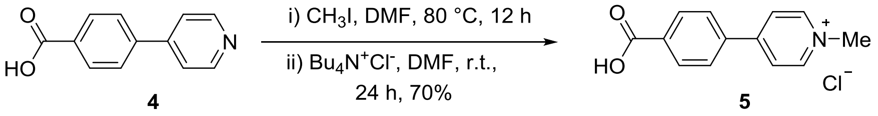

Compound 5. A mixture of compounds **4** (0.20 g, 1.0 mmol) and methyl iodide (0.71 g, 5.0 mmol) in DMF (20 mL) was stirred at 80 °C for 12 h and then added tetrabutylammonium chloride (2.78 g, 10.0 mmol). After the mixture was cooled to r.t, the formed precipitate was filtrated, washed with DMF (20 mL), and dried under vacuum to afford **5** as a pale white solid (0.18 g, 70%). M.p. > 300 ℃ (decomp). ^{1}H NMR (400 MHz, DMSO-d$_6$): δ 13.37 (s, 1H), 9.08 (d, *J* = 6.8 Hz, 2H), 8.56 (d, *J* = 6.9 Hz, 2H), 8.20-8.14 (m, 4H), 4.36 (s, 3H). ^{13}C NMR (100 MHz, DMSO): δ 167.04, 153.74, 146.32, 137.97, 133.98, 130.74, 128.88, 125.18, 47.78. MS (ESI): *m/z* 214.1 $[M-Cl]^+$. HRMS: Calcd for $C_{13}H_{12}NO_2$ $[M-Cl]^+$: 214.0863. Found: 214.0858.

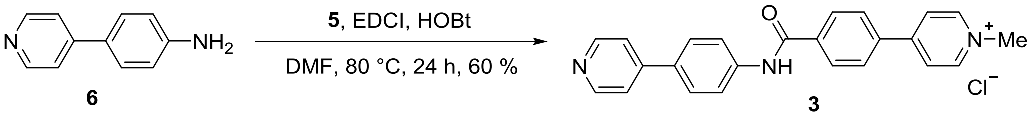

Compound 3. A mixture of compounds **6** (0.17 g, 1.0 mmol), **5** (0.28 g, 1.1 mmol), EDCI (0.21 g, 1.1 mmol) and *N*-hydroxybenzotrizole (HOBt, 0.15 g, 1.1 mmol) in DMF (20 mL) was stirred at 80 °C for 24 h and then cooled to r.t, the formed precipitate was filtrated, washed with DMF (20 mL), and dried under vacuum to afford **3** as an yellow solid (0.24 g, 60%). M.p. > 300 ℃ (decomp). ^{1}H NMR (400 MHz, DMSO-d$_6$): δ 10.85 (s, 1H), 9.13 (d, *J* = 6.8 Hz, 2H), 8.63-8.61 (m, 4H), 8.31-8.25 (m, *J* = 8.7 Hz, 4H), 8.05 (d, *J* = 8.7 Hz, 2H), 7.87 (d, *J* = 8.7 Hz, 2H), 7.74 (d, *J* = 6.4 Hz, 2H), 4.38 (s, 3H). ^{13}C NMR (100 MHz, DMSO-d$_6$): δ 165.15, 153.67, 150.68, 146.86, 146.34, 140.75, 137.73, 136.78, 132.59, 129.59, 128.58, 127.50, 124.99, 121.41, 121.16, 47.64. MS (ESI): *m/z* 366.2 $[M-Cl]^+$. HRMS (ESI): Calcd for $C_{24}H_{20}N_3O$: 366.1601 $[M-Cl]^+$. Found: 366.1601.

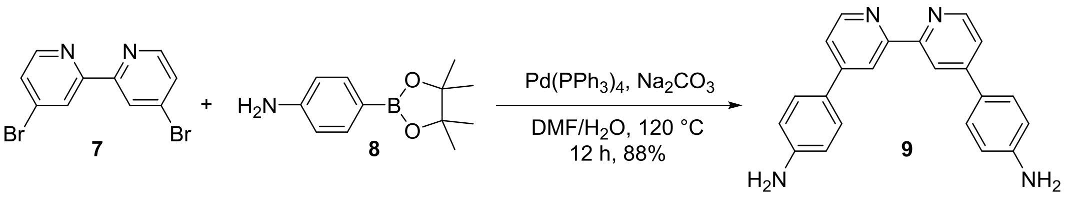

Compound 9. A mixture of compounds **7** (0.31 g, 1.0 mmol), **8** (0.48 g, 2.2 mmol), tetrakis(triphenylphosphine)palladium (58 mg, 1.1 mmol) and Na_2CO_3 (0.53 g, 5.0 mmol) in DMF (10 mL) and H_2O (5 mL) was stirred at 120 °C for 12 h under and then H_2O (30 mL) was added. The mixture was cooled to r.t, the formed precipitate was filtrated, washed with H_2O (30 mL), and dried under vacuum to afford **9** as a pale yellow solid (0.70 g, 88%). M.p. > 300 ℃ (decomp). ^{1}H NMR (400 MHz, DMSO-d$_6$): δ 8.69-8.51 (m, 4H), 7.70-7.55 (m, 6H), 6.70 (d, *J* = 8.5 Hz, 4H), 5.58

(s, 4H). ^{13}C NMR (100 MHz, DMSO): δ 155.84, 150.40, 149.65, 148.29, 127.54, 123.55, 119.73, 115.88, 114.15. MS (ESI): *m/z* 339.2 $[M+H]^+$. HRMS: Calcd for $C_{22}H_{19}N_4$ $[M+H]^+$: 339.1604. Found: 339.1590.

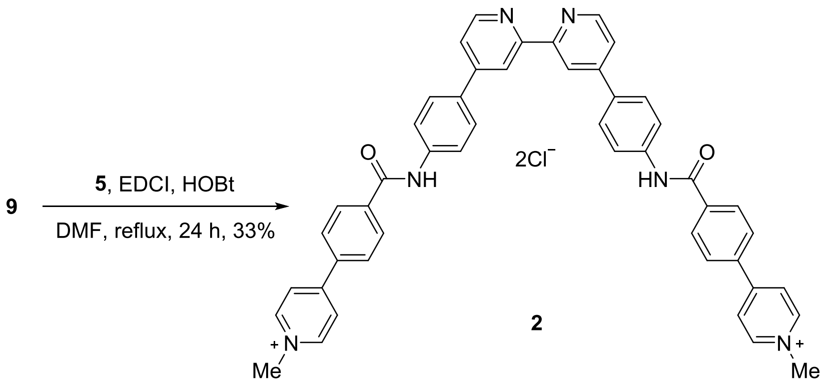

Compound 2. A mixture of compounds **5** (0.17 g, 0.5 mmol), **9** (0.28 g, 1.1 mmol), EDCI (0.21 g, 1.1 mmol) and *N*-hydroxybenzotrizole (HOBt, 0.15 g, 1.1 mmol) in DMF (20 mL) was stirred at reflux for 24 h. After cooling to r.t, the formed precipitate was filtrated, washed with DMF (20 mL), and dried under vacuum to afford **2** as an orange solid (0.13 g, 33%). M.p. > 300 ℃ (decomp). ^1H NMR (400 MHz, DMSO-d_6): δ 10.71 (s, 2H), 9.10 (d, *J* = 6.7 Hz, 4H), 8.81 (d, *J* = 5.0 Hz, 2H), 8.77 (s, 2H), 8.62 (d, *J* = 6.8 Hz, 4H), 8.37 – 8.19 (m, 8H), 8.07 (d, *J* = 8.6 Hz, 4H), 7.98 (d, *J* = 8.7 Hz, 4H), 7.86 (d, *J* = 5.1, 2H), 4.38 (s, 6H). ^{13}C NMR (100 MHz, DMSO-d_6): δ 164.66, 155.91, 153.21, 150.04, 147.64, 145.80, 140.25, 137.36, 136.35, 132.35, 128.94, 128.15, 127.26, 124.53, 121.22, 120.89, 117.28, 47.20. MS (ESI): *m/z* 365.2 $[M-2Cl]^{2+}$. HRMS (ESI): Calcd for $C_{48}H_{38}N_6O_2$: 365.1523 $[M-2Cl]^{2+}$. Found: 365.1539.

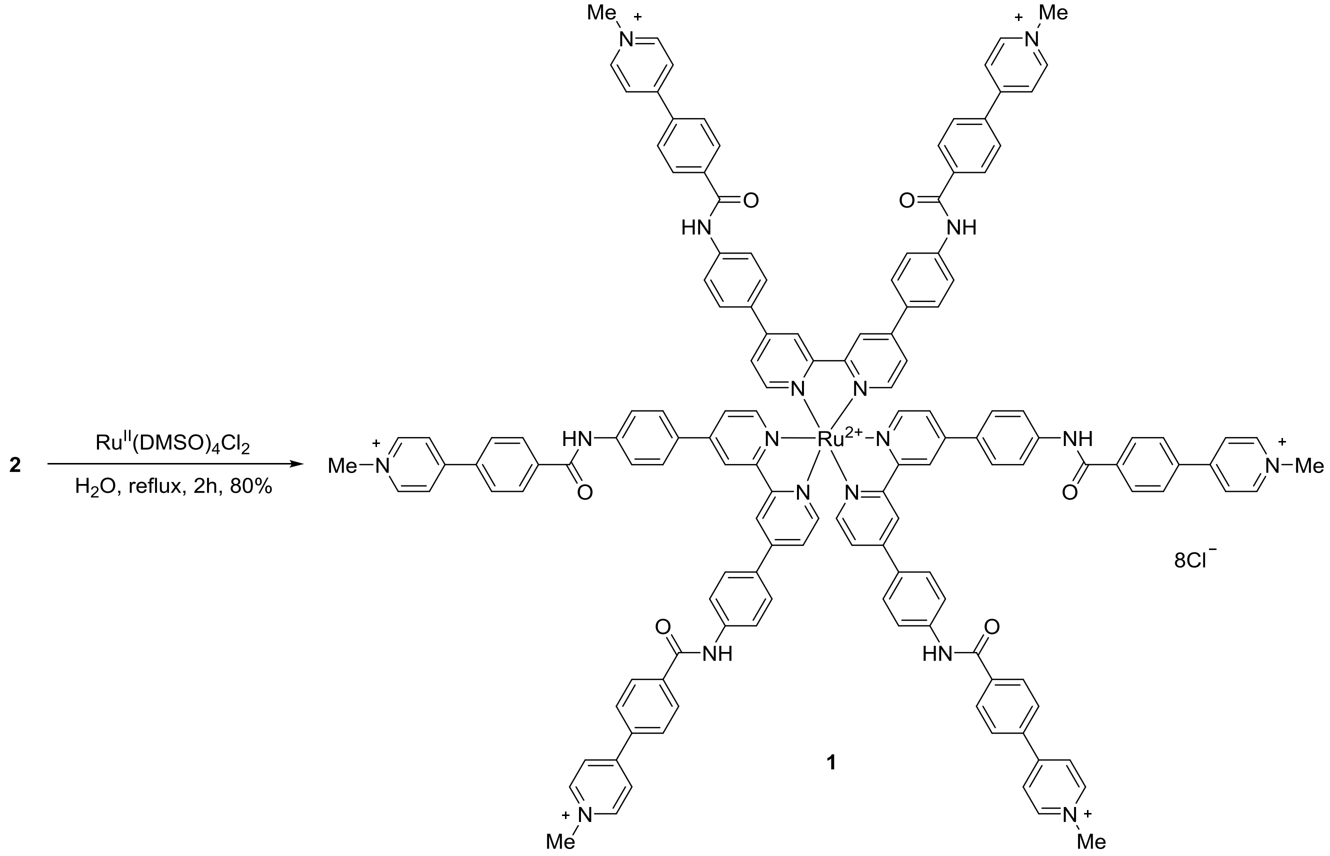

Compound 1. A mixture of compounds **2** (26.4 mg, 0.033 mmol) and tetrakis(dimethylsulfoxide)dichlororuthenium (4.8 mg, 0.01 mmol) in water (5 mL) was stirred under reflux for 2 h and then cooled to room temperature. The solvent was evaporated under reduced pressure and the resulting red solid was washed by acetonirile (5 mL) and dried under vacuum. The solid was further recrystallized from water and DMF (2:1) to give compound **1** as a dark red solid (20.6 mg, 80%). M.p. > 300 ℃ (decomp). ^{1}H NMR (400 MHz, DMSO-d_6) δ 10.70 (s, 6H), 9.09 (d, *J* = 7.0 Hz, 12H), 8.78 (m, 12H), 8.61 (d, *J* = 6.8 Hz, 12H), 8.32-8.19 (m, 24H), 8.06 (d, *J* = 8.6 Hz, 12H), 7.98 (d, *J* = 8.7 Hz, 12H), 7.86-7.85 (m, 6H), 4.37 (s, 18H). ^{13}C NMR (100 MHz, DMSO-d_6): δ 165.11, 156.34, 153.62, 150.49, 148.08, 146.26, 140.73, 137.76, 136.78, 132.76, 129.42, 128.60, 127.69, 124.96, 121.67, 121.35, 117.72, 47.64. HRMS (ESI): Calcd for $C_{144}H_{114}N_{18}O_6Ru$: 286.6022 $[M-8Cl]^{8+}$. Found: 286.6030. Elemental analysis calcd for $C_{144}H_{114}N_{18}O_6Ru$ (%): C, 75.41; H, 5.01; N, 10.99; Found: C 74.87, H 5.07, N 10.53.

The method for the determination of the apparent association constants. The concentration of CB[8] and the concentration of the 4-phenylpyridin-1-ium (PhPy) unit of the investigated compounds were kept at 1:2. The thermodynamics of the host-guest complexation and competition experiments were defined as the following equations[4].

$$CB[8] + 2PhPy \underset{}{\overset{K_a}{\rightleftharpoons}} CB[8]\subset 2PhPy \quad (1)$$

CB[8] + 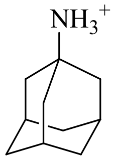 $\overset{K_{AD}}{\rightleftharpoons}$ CB[8]⊂AD (2)

AD

$$CB[8]\subset 2PhPy + AD \overset{K_{rel}}{\rightleftharpoons} CB[8]\subset AD + 2PhPy \quad (3)$$

For compounds **1** and **2**, their complexes with CB[8] exhibited a set of new signals. The following equation was used to determine K_a:

$$K_{rel} = 4C^0_{CB[8]\subset 2PhPy} \frac{x^3_{free}}{(1 - x_{free})\left(C^0_{AD}/C^0_{CB[8]\subset 2PhPy} - x_{free}\right)} \quad (4)$$

Where $K_{rel} = K_{AD}/K_a$, $C^0_{CB[8]\subset 2PhPy}$ was the concentration of the 2:1 complex of PhPy and CB[8], C^0_{AD} was the total concentration of guest 1-adamantanamine hydrochloride, x_{free} was the ratio of the concentration of the free PhPy unit over the total concentration of the PhPy unit.

For compound **3** the chemical shifts of its signals changed upon complexation by CB[8]. The following equations were used to determine K_a:

$$\delta = \delta_{free} \times x_{free} + \delta_{complex} \times x_{complex} \quad (5)$$

$$K_{rel} = 4C^0_{CB[8]\subset 2PhPy} \frac{\left(\frac{\delta - \delta_{complex}}{\delta_{free} - \delta_{complex}}\right)^3}{\left(\frac{\delta_{free} - \delta}{\delta_{free} - \delta_{complex}}\right)\left(C^0_{AD}/C^0_{CB[8]\subset 2PhPy} - \frac{\delta - \delta_{complex}}{\delta_{free} - \delta_{complex}}\right)} \quad (6)$$

Where $K_{rel} = K_{AD}/K_a$, $C^0_{CB[8]\subset 2PhPy}$ was the concentration of the 2:1 complex of the PhPy unit and CB[8], C^0_{AD} was the concentration of 1-adamantanamine hydrochloride, $\delta_{complex}$ represents the chemical shifting of the signal of the PhPy unit in the complex, δ_{free} represents the chemical shifting of the signal of the free PhPy unit, x_{free} was the ratio of the concentration of the free PhPy unit, $x_{complex}$ was the ratio of the concentration of the PhPy unit in the complex. K_{AD} had been previously determined to be 8.2×10^8 M^{-1} in 50 mM CD_3CO_2Na buffer (pD = 4.74).

Dynamic light scattering (DLS) measurement. DLS data were obtained on a Malvern Zetasizer Nano ZS90 using a monochromatic coherent He–Ne laser (633 nm) as the light source and a detector that detected the scattered light at an angle of 90 °.

TGA measurement. TGA experiments were performed on a Model TGA/SDTA 851 instrument. Samples were placed in alumina pans and heated at a rate of 5 ℃ per minute from 30 to 800 ℃ under a nitrogen atmosphere.

Gas adsorption experiments. Gas adsorptions of N_2 at 196 °C and CO_2 at 0 °C were measured on a Micromeretics Model ASAP 2020 gas adsorption analyzer. About 43.5 mg of activated sample was degassed at 150 °C for 12 h by using the "outgas" function of the surface area analyzer. Helium gas was used to estimate the dead volume. The saturation pressure (P_0) was measured throughout the N_2 analyses via a dedicated saturation pressure transducer, which helped to monitor the vapor pressure for each data point. Part of the N_2 sorption isotherm in the normalized pressure (P/P_0) range of 0.05−0.3 was used to calculate the BET surface area. For CO_2 isotherm measurements, activated sample was transferred into a pre-weighed glass sample tube. The tube was then sealed and quickly transferred to a system providing 10^{-4} torr dynamic vacuum. The sample was kept under this vacuum at 150 °C for 12 h and then used for CO_2 adsorption measurements.

V**isible light-driven H_2 production. Photocatalytic H_2 production in the aqueous solution:** The photocatalytic water reduction was carried out in an external illumination type reaction vessel with a magnetic stirrer. Samples for photocatalytic hydrogen production were prepared in 5 mL septum-sealed glass vials. Each sample was made up to a volume of 2.2 mL 10% methanol (v:v) aqueous solution with the pH value of 1.8 (adjusted by 2 M HCl). Samples typically contained 0.03 to 3 mM of **1** and 0.002 to 0.6 mM of **WD-POM**. Sample vials were capped and deoxygenated by bubbling nitrogen through them for 30 min to ensure complete air removal. The solution was irradiated by a 300 W solid state light source with a 500 nm filter. After the hydrogen evolution reaction, the gas in the headspace of the vial was analyzed by GC to determine the amount of hydrogen generated.

Photocatalytic H_2 production in the DMF/CH_3CN mixed solution: Samples for photocatalytic hydrogen production were prepared in 5 mL septum-sealed glass vials. Each sample was made up to a volume of 2 mL with appropriate triethanolamine or methanol in DMF/CH_3CN (7:3 v/v) solution containing 200 μL pH 2.4 water (adjusted by 2 M HCl). Samples typically contained 0.03 to 3 mM of **1** and 0.002 to 0.6 mM of **WD-POM**. Sample vials were capped and deoxygenated by bubbling nitrogen through them for 30 min to ensure complete air removal. The solution was irradiated by a 300 W solid state light source with a 500 nm filter. After the hydrogen evolution reaction, the gas in the headspace of the vial was analyzed by GC to determine the amount of hydrogen generated.

UV-vis absorption experiments for detecting the possible leaching of WD-POM from WD-POM@SMOF-1 during the H_2 production: (i) For the homogeneous system, the H_2 production reaction was carried out in a transparent dialysis tubing (Spectra/Por 6 Dialysis Tubing, 10 kDa Molecular Weight Cut Off, 8 mm Flat-width). The dialysis tubing was placed in a 20 mL reaction vessel with a magnetic stirrer, containing 12 mL water/MeOH (10:1, v/v, pH = 1.8 adjusted by HCl). A solution of **WD-POM@SMOF-1** in 2.2 mL water/MeOH (10:1 v/v, pH = 1.8 adjusted by HCl, [**1**] = 0.3 mM, [**WD-POM**] = 0.02 mM) was injected with a syringe into the dialysis tubing. After deoxygenation, the solution was irradiated, as described above, for 50 hours for H_2 production. The UV-vis spectrum of the solution was then recorded every 5 hours. (ii) For the heterogeneous system, the solid sample was obtained by evaporation of a 2.2 mL solution of

WD-POM@SMOF-1 ([**1**] = 0.3 mM, [**WD-POM**] = 0.02 mM) in water in a 5 mL septum-sealed glass vial. To the vial was added a 2.3 mL solution of DMF/MeCN/triethnaolamine/water (14:6:1:2, v/v/v/v, pH = 2.4 adjusted by HCl). After deoxygenation, the suspension was irradiated, as described above, for 50 hours for H_2 production. The UV-vis spectrum of the supernatant solution was then recorded every 5 hours. The supernatant was obtained by centrifugation (8000 r/min) of the sample for 5 minutes to remove suspended solids. For both the homogeneous and heterogeneous systems, to the limit of the instrument (absorbance: 0.01 a.u.), the spectra (270 nm-700 nm) did not exhibit any detectable absorbance of **WD-POM** or **SMOF-1** after irradiation for 50 hours. For comparison, the UV-vis spectra of **WD-POM** (0.02 mM) in the two solvents were also recorded, both of which exhibited strong absorption in the wavelength range (see Supplementary Figures 30b and 30c).

The Crystallite Size Calculation. The average crystallite sizes of **SMOF-1** and **WD-POM@SMOF-1** before and after irradiation for 20 hours were estimated to be both about 50 nm, 53 nm and 60 nm from the XRD results (Figures 3d and 3f and Supplementary Figure 25) using the Debye-Scherrer equation, $D = K\lambda / (\beta\cos\theta)$, where D is the average crystal diameter, β is the corrected peak width (full width at half maximum), K is a constant related the shape of the crystallites (K=0.9), λ is the X-ray wavelength of Cu Kα radiation (1.54 Å), and θ is the diffraction angle. The width of the diffraction peak with the highest intensity was selected for the calculation.

Supplementary References

[1]. Gao, B., et al. Starburst substituted hexaazatriphenylene compounds: Synthesis, photophysical and electrochemical properties. *Tetrahedron Lett.* **50**, 1649-1652 (2009).

[2]. Zhang, Z. M. et al. Photosensitizing metal-organic framework enabling visible-light-driven proton reduction by a wells-dawson-type polyoxometalate. *J. Am. Chem. Soc.* **137**, 3197–3200 (2015).

[3]. Graham, C. R. & Finke, R. G. The classic wells−dawson polyoxometalate, $K_6[\alpha\text{-}P_2W_{18}O_{62}]\cdot 14H_2O$. Answering an 88 year-old question: What is its preferred, optimum synthesis? *Inorg. Chem.* **47**, 3679-3686 (2008).

[4]. Liu, S. *et al.* The cucurbit[n]uril family: prime components for self-sorting systems. *J. Am. Chem. Soc.* **127**, 15959–15967 (2005).
